# Seed-mediated synthesis of NHC-stabilised Cu@Au core–shell nanoparticles from an NHC-Au(i) complex

**DOI:** 10.1039/d6nr01169a

**Published:** 2026-06-23

**Authors:** Monnaya Chalermnon, Robert Richstein, Janine Lichtenberger, Domenico Grammatico, Lingcong Ge, Rachmat Adhi Wibowo, Jia Min Chin, Michael R. Reithofer

**Affiliations:** a Institute of Inorganic Chemistry, Faculty of Chemistry, University of Vienna Währinger Str. 42 1090 Vienna Austria michael.reithofer@univie.ac.at; b Center for Energy, Power and Renewable Gas Systems, AIT Austrian Institute of Technology GmbH Giefinggasse 2 1210 Vienna Austria; c Institute of Functional Materials and Catalysis, Faculty of Chemistry, University of Vienna Währinger Str. 42 1090 Vienna Austria jiamin.chin@univie.ac.at; d Wolfgang Pauli Institute, Oskar-Morgenstern-Platz 1 1090 Wien Austria

## Abstract

N-heterocyclic carbenes (NHCs) provide a robust platform for the stabilisation and functionalisation of metal nanoparticles. Extending this ligand class to air-sensitive copper-based nanomaterials is particularly attractive, but remains challenging because copper nanoparticles readily oxidise under ambient conditions. Here, we report the seed-mediated synthesis of 9 nm copper–gold core–shell nanoparticles (CSNPs) with a ∼2 nm gold shell, prepared by reducing a NHC-Au(i) complex on pre-formed copper nanoparticle seeds. The resulting samples, denoted as IC12@CSNP and IC12@CSNP_NHC_, were characterised by X-ray Photoelectron Spectroscopy (XPS) and Surface-Enhanced Raman Spectroscopy (SERS), confirming surface binding of IC12 ligands. Scanning Transmission Electron Microscopy (STEM) and High-Resolution Transmission Electron Microscopy (HRTEM) verified the distinct gold shell and copper core. The gold shell helps preserve the metallic character of the copper core upon exposure to air. Both IC12@CSNP and IC12@CSNP_NHC_ exhibit electrocatalytic activity for syngas production under CO_2_ reduction conditions, with the H_2_ : CO ratio tunable from 0.9 : 1 to 2.7 : 1 by varying the applied potential. This work establishes a straightforward synthetic route to air-stable, NHC-functionalised copper–gold core–shell nanoparticles with well-defined structure and tunable catalytic behaviour.

## Abstract 2

A seed-mediated, bottom-up synthesis of NHC-protected Cu@Au core–shell nanoparticles is reported. The resulting NHC@CuAuNPs show remarkable ambient stability and promise as electrocatalysts for CO_2_RR-driven syngas production.

## Graphical abstract

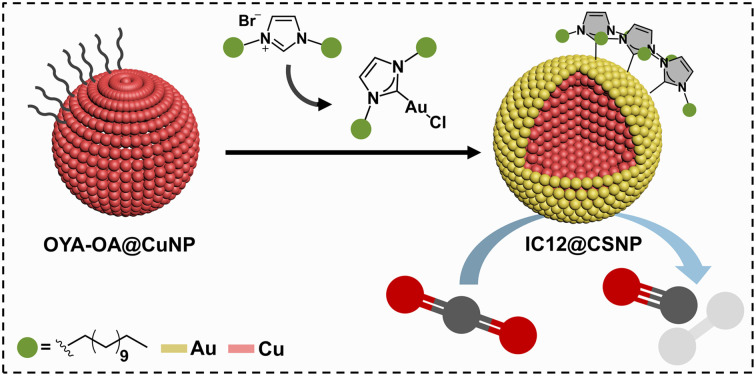

## Introduction

N-heterocyclic carbenes (NHCs) have been established as robust functionalising and stabilising agents for metal nanoparticles, offering versatile alternatives to the thiol or phosphine ligands.^1,2^ Numerous studies have explored NHC-functionalised monometallic nanoparticles, including NHC-Ru,^3^ NHC-Pt,^4^ NHC-Cu,^5^ and NHC-Au.^6–8^ Among these, NHC-Au systems are one of the most extensively studied, owing to their exceptional stability in organic solutions, biological media, or as self-assembled monolayers.^9,10^ Beyond stability, NHC ligands can enhance catalytic performance, such as in electrochemical CO_2_ reduction (CO_2_RR). For instance, gold nanoparticles (AuNPs) capped with sterically demanding NHC ligands achieved a Faradaic efficiency (FE) for CO of 83% at −0.57 V *vs.* RHE, outperforming oleylamine-capped gold nanoparticles. Similarly, polymeric NHC-Au nanoparticles exhibited 90% FE (−0.9 V *vs.* RHE) compared to unmodified gold nanoparticles.^8,11^

Copper nanoparticles offer the unique and desirable advantage of reducing CO_2_ into C_2+_ products; however, they often exhibit poor product selectivity, are inherently prone to the competing Hydrogen Evolution Reaction (HER), and are highly susceptible to oxidation under ambient conditions, all of which result in diminished electrocatalytic performance.^12^ Approaches to overcome copper oxidation include the use of organic capping agents, surface modification, and a protective shell layer.^13^ In this regard, we recently demonstrated the synthesis of NHC@CuNPs from easily accessible NHC-Cu complexes.^5^ Although these compounds displayed excellent stability under inert conditions, the NHC ligands were unable to stabilise the CuNPs under air, resulting in their readily oxidation and the release of imidazolium ligands.

An alternative strategy to prevent CuNPs oxidation is the use of a metallic shell layer, which can be achieved through electroplating or electroless plating (*i.e.*, the use of a reducing agent).^14^ Recent examples demonstrated the synthesis of copper nanowires coated with different metals, such as silver,^14,15^ gold,^16,17^ or nickel.^18^ In terms of nanoparticles, Ma and coworkers reported the synthesis of ultra-stable plasmonic Cu–Au core–shell nanoparticles through a carefully controlled galvanic replacement process, where an Au^3+^ precursor is replaced onto a copper nanoparticle core using trioctylphosphine as a stabilising ligand. Although NHC@AuNPs have shown higher stability and chemical inertness compared to thiol-based analogues, there are no reports on the synthesis of NHC-protected Cu–Au core–shell particles. In general, reports on bimetallic NHC-stabilised nanoparticles are limited; for example, Nazemi and co-workers recently reported the first synthesis of poly-(N-heterocyclic carbene)-capped AuAg bimetallic nanoparticles through the reduction of polymeric NHC-AuAg complex with *tert*-butylamine borane complex.^19^ It was found that the ratio of gold and silver in the copolymer influenced and dictated whether alloy or core–shell nanoparticles were fabricated. Other examples include water-soluble NHC-stabilised mono- and bimetallic Pd/Ni nanoparticles for H/D exchange reactions,^20^ and NHC-stabilised Au–Pd alloy nanoparticles as a biomimetic catalyst and a heterogeneous catalyst.^21^

**Fig. 1:**
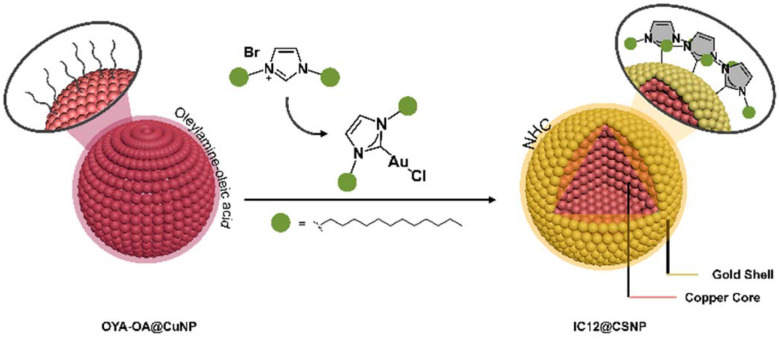
General synthesis scheme of core–shell nanoparticles involving OYA-OA@CuNP as seed, which [Au(IC12)Cl] was reduced through a bottom-up method to yield IC12@CSNP and IC12@CSNP_NHC_.

Herein, we report a facile synthesis of 1,3-didodecylimidazolium (IC12) functionalised copper–gold core–shell nanoparticles, through the reduction of [Au(IC12)Cl] complex onto oleylamine-oleic acid stabilised copper nanoparticles (OYA-OA@CuNP) (Fig. 1). The resulting NHC-stabilised Cu–Au core–shell nanoparticles are air-stable. The binding of the NHC ligand onto the Au layer was proven through X-ray Photoelectron Spectroscopy (XPS) and Surface-enhanced Raman spectroscopy (SERS). Further detailed Scanning Transmission Electron Microscopy (STEM) and High-Resolution Transmission Electron Microscopy (HRTEM) analysis were utilised to verify the formation of a core–shell structure rather than an alloy. Finally, we demonstrate that these core–shell particles can be utilised for syngas production through CO_2_RR, whereby the H_2_ : CO ratio can be fine-tuned by varying the applied potential.^16,22^

## Results and discussion

### Synthesis and characterisation of core–shell nanoparticles

The core–shell nanoparticles were obtained based on an adapted seed-mediated procedure. First, copper nanoparticles (CuNP) were prepared as ‘seeds’ using CuBr(PPh_3_)_3_ as the copper source and a mixture of oleylamine (OYA) and oleic acid (OA) at a 1.25 : 1 ratio as the stabilising agents.^23^ The OYA-OA@CuNP were characterised by TEM and XPS (Fig. S1 and S2, in the SI). The gold shell was formed by reducing the [Au(IC12)Cl] gold source with *tert*-butylamine borane (TBAB) onto the OYA-OA@CuNP, yielding IC12@CSNP.^24^ Alternatively, the core–shell particles were also synthesised in the presence of additional free carbene. For this, free carbene prepared by deprotonation of 1,3-didodecylimidazolium bromide (IC12Br) with potassium bis(trimethylsilyl)amide (KHMDS) was added to the reaction mixture of OYA-OA@CuNP and [Au(IC12)Cl], yielding IC12@CSNP_NHC_. Once the reactions were complete, IC12@CSNP and IC12@CSNP_NHC_ were purified outside the glovebox, by washing with acetone and ethyl acetate, followed by dispersing in toluene, yielding a deep red solution at ambient conditions. Further, it is worth noting that both core–shell particles can withstand multiple drying and redispersion cycles in toluene without any visible changes. For comparative purposes, oleylamine functionalised gold nanoparticles (OYA@AuNP) and IC12 functionalised gold nanoparticles (IC12@AuNP) were synthesised using the reported procedures through the bottom-up approach.^25,26^

**Fig. 2:**
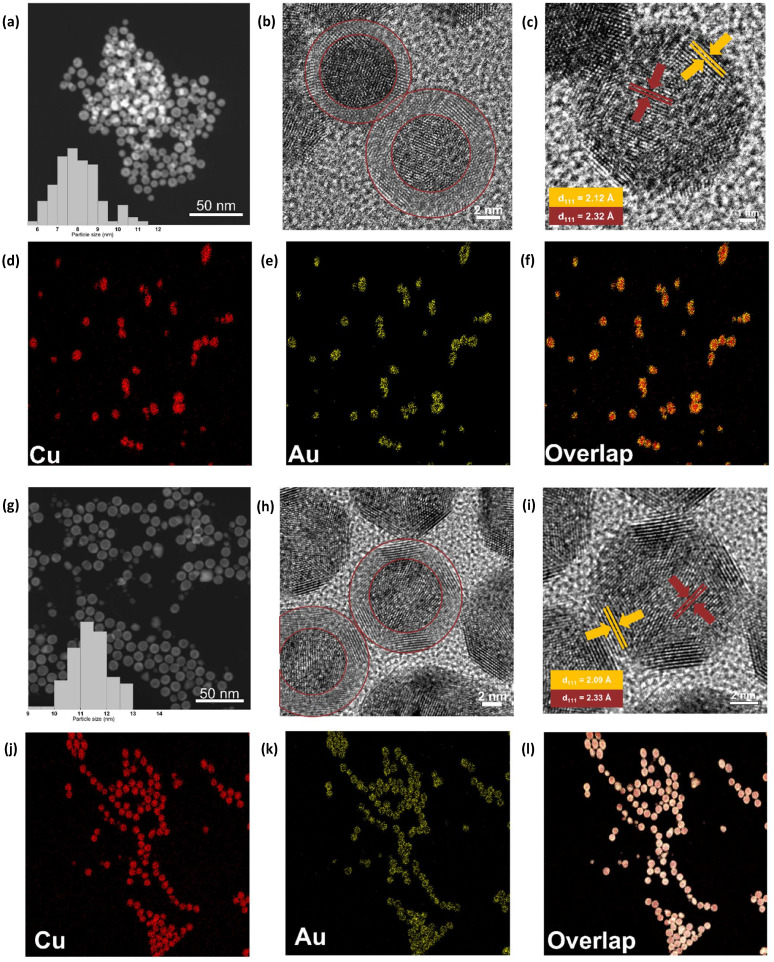
(a) STEM, (b–c) HRTEM, and (d–f) STEM-EDS images of IC12@CSNP; (g) STEM, (h–i) HRTEM, and (j–l) STEM-EDS of IC12@CSNP_NHC_. The red-labelled *d*-spacing is Cu(111) and the yellow-labelled *d*-spacing is Au(111).

The morphology and size of all nanoparticles were examined using STEM and HRTEM (Fig. 2 and Fig. S1 and S3–S4). STEM images of IC12@CSNP and IC12@CSNP_NHC_ confirmed the core–shell structure, with the heavier gold element displaying bright signals, and the lighter copper element as the dark core. The similar sizes of IC12@CSNP (9.4 ± 1 nm) and IC12@CSNP_NHC_ (9.2 ± 1 nm) indicated that the inclusion of free carbene during the synthesis had minimal effect on nanoparticle size. HRTEM images further verified the core–shell structure through distinct *d*-spacing patterns (Fig. 2b, c and h, i), where the copper core exhibited characteristic Cu(111) *d*-spacings of 2.09 Å and 2.12 Å for IC12@CSNP and IC12@CSNP_NHC_.^27^ The gold shell was confirmed by the characteristic Au(111) *d*-spacing of 2.32 Å for IC12@CSNP and 2.33 Å for IC12@CSNP_NHC_.^28^ Additionally, the gold shell thickness was measured with HRTEM, showing that IC12@CSNP and IC12@CSNP_NHC_ exhibited relatively similar shell thicknesses of 2.1 ± 0.3 nm and 2.0 ± 0.3 nm, respectively. It should be noted that CuAu alloying is possible at the interface between the core and the shell, but it was likely minimal at the current synthesis temperature. To further verify the core–shell structure, Energy Dispersive X-ray Spectroscopy (EDS) and line-scan EDS were employed. EDS images clearly showed the core–shell conformation, with copper at the center surrounded by a gold shell (Fig. 2d–f and j–l). Additionally, line-scan EDS showed that gold counts remained constant across the diameter, while copper counts increased towards the nanoparticle center (Fig. S5). Lastly, STEM of IC12@AuNP shows that the nanoparticles were in the same particle size range as the core–shell nanoparticles at 8.2 ± 1 nm (Fig. S3).

**Fig. 3:**
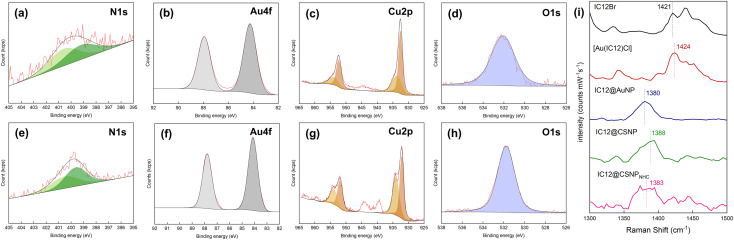
N 1s, Au 4f, Cu 2p, and O 1s XPS spectra of (a–d) IC12@CSNP, (e–h) IC12@CSNP_NHC_, (i) Raman spectra of IC12Br (black line), [Au(IC12)Cl] (red line), IC12@AuNP (blue line), IC12@CSNP (green line), and IC12@CSNP_NHC_ (pink line).

The presence of organic components on the nanoparticle was confirmed by thermogravimetric analysis (TGA), which showed a weight loss between 250 °C and 400 °C (Fig. S6). IC12@CSNP_NHC_ possessed 3% higher organic content than IC12@CSNP, indicating that the additional free carbene was incorporated onto the nanoparticles. The ligand coverage was calculated from TGA data, revealing that IC12@CSNP and IC12@CSNP_NHC_ contained 0.56 and 0.64 NHC ligands per Au atom, respectively. XPS analysis confirmed that the NHC ligands are surface-bound to the nanoparticles, as indicated by the distinct N 1s binding energy of the carbene nitrogen. The imidazolium salt typically exhibits a binding energy above 401.8 eV, while the surface-bound NHC has a lower binding energy at 399–401 eV.^5,29^ As shown in the N 1s XPS spectra, two peaks were observed at 399.2 eV and 400.6 eV for IC12@CSNP, and 399.6 and 400.5 eV for IC12@CSNP_NHC_, indicating the C–N=C and the N–C^2^ contributions (Fig. 3a and e).^30^ Similarly, the N 1s XPS of IC12@AuNP exhibits two peaks at 399.8 and 401.3 eV, indicating NHC functionalisation on the gold nanoparticles (Fig. S7). By leveraging the SERS properties of the gold and copper nanoparticles, the N–C=N symmetric stretching frequency of the imidazolium salt, imidazolium gold complex, and NHC-bounded nanoparticles were measured. A typical red shift from 1424 cm^−1^ for [Au(IC12)Cl] to 1388, 1383, and 1380 cm^−1^ for IC12@CSNP, IC12@CSNP_NHC_ and IC12@AuNP, respectively, was observed, which is attributed to the change of the electron density of NHC's π* orbital caused by π-backbonding of the metal to NHCs (Fig. 3i and Fig. S8).^31^ Similar red shifts have been observed in several cases of imidazolium- and benzimidazolium-functionalised gold nanoparticles and gold film-over-nanosphere (Au-FON) substrates.^31,32^

The Au and Cu oxidation states were also examined using XPS. Both core–shell nanoparticles exhibited a gold outer shell composed only of Au^0^. The Au 4f binding energies were measured at 84.3 eV (Au 4f_7/2_) and 87.9 eV (Au 4f_5/2_) for IC12@CSNP, and 84.1 eV and 87.8 eV for IC12@CSNP_NHC_ (Fig. 3b and f). The observed spin–orbit coupling shift of 3.67 eV is characteristic of the Au^0^ oxidation state.^24^ Interestingly, no evidence of Au^1+^ was found after careful fitting of the Au 4f XPS spectra, although the bottom-up synthesis of NHC@AuNPs often results in a mixed oxidation state of gold nanoparticles.^6,33^ We attribute these findings to a galvanic redox process between Cu (standard reduction potential: 0.34 V, Cu^2+^/Cu^0^) and Au (standard reduction potential: 1.69 V, Au^1+^/Au^0^), which facilitates the complete reduction of Au^1+^ to Au^0^.^34,35^ Through this galvanic process, partial oxidation of the copper core was observed by XPS, resulting in a mixed oxidation state (Fig. 3c and g).^36^ The Cu 2p XPS spectra of IC12@CSNP displayed binding energies at 932.6 eV (Cu 2p_2/3_) and 952.4 eV (Cu 2p_1/2_), which cannot be clearly assigned to one copper oxidation state and most likely correspond to either Cu^0^ and/or Cu^1+^. However, peaks at 933.7 eV (Cu 2p_2/3_) and 953.9 eV (Cu 2p_1/2_) can be assigned to the Cu^2+^ oxidation state.^37^ For IC12@CSNP_NHC_, similar binding energies were observed in the Cu 2p XPS spectra at 932.1/951.9 eV and 934.0/954.0 eV for Cu^0^/Cu^1+^ and Cu^2+^, respectively. Using the Cu Auger Spectra, Cu^0^ with the characteristic binding energy of 568.0 eV is differentiated from Cu^1+^ at 570 eV.^38^ The Auger spectra indicated that IC12@CSNP contained predominantly Cu^0^ at 567.9 eV, while IC12@CSNP_NHC_ displayed a slight shift of binding energy for Cu^0^ at 567.6 eV (Fig. S9). Notably, the Cu^2+^ species was more pronounced in IC12@CSNP_NHC_ than IC12@CSNP, as evidenced by the two characteristic satellite peaks at around 940 eV and 944 eV, attributed to the ‘shake up’ process of the electrons.^39^ The O 1s XPS further supported the presence of Cu^1+^ or Cu^2+^ species, as the binding energy for oxide species was observed at 532.1 eV and 531.8 eV for IC12@CSNP and IC12@CSNP_NHC_, respectively (Fig. 3d and h). Despite the higher content of Cu^2+^ in IC12@CSNP_NHC_, a study has suggested that it should not hinder the CO_2_RR activity, as oxidised copper could partially reduce to metallic copper under the electrochemical reduction conditions.^40,41^

Quantifications of the gold/copper content of the core–shell nanoparticles were conducted using Inductively Coupled Plasma Mass Spectrometry (ICP-MS). A ratio of gold to copper was found to be 3.2 : 1 (Au : Cu) for IC12@CSNP, and 2.1 : 1 for IC12@CSNP_NHC_. Lastly, the UV-Vis spectra of core–shell nanoparticles showed the combined Localised Surface Plasmon Resonance (LSPR) characteristic bands of copper and gold nanoparticles, revealing peak maxima of IC12@CSNP at 555 nm and IC12@CSNP_NHC_ at 572 nm (Fig. S10).^35^ The absorbance peaks of OYA@AuNP and IC12@AuNP were observed at 529 nm and 537 nm, corresponding to the LSPR band of gold nanoparticles (Fig. S11).^25,29^ In addition, the stability of all synthesised nanoparticles was monitored using UV-Vis spectroscopy. For the core–shell nanoparticles, a decrease in absorbance intensity was observed over 24 hours due to nanoparticle aggregation and sedimentation; however, the nanoparticles can be redispersed after sonication, resulting in a strong LSPR signal again.

### Electrochemical CO_2_RR of nanoparticles

**Fig. 4:**
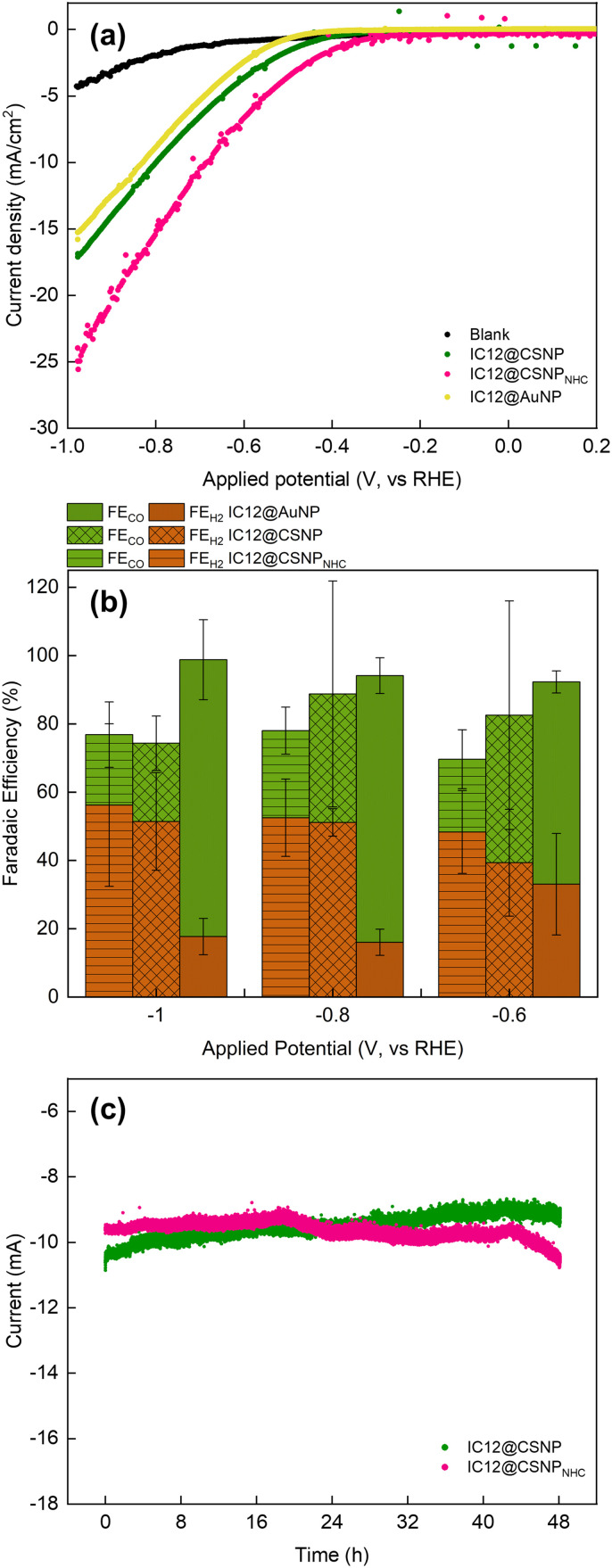
(a) Linear sweep voltammograms of IC12@CSNP, IC12@CSNP_NHC_, IC12@AuNP, and blank in CO_2_-purged 0.5 M KHCO_3_; (b) Faradaic efficiency of CO and H_2_ for IC12@CSNP, IC12@CSNP_NHC_, and IC12@AuNP; (c) chronoamperometry measurement for the 48-hour stability test of IC12@CSNP and IC12@CSNP_NHC_.

The synthesised catalysts were evaluated for their CO_2_RR catalytic performance. The experiments were conducted in an H-cell containing a CO_2_-purged 0.5 M KHCO_3_ electrolyte. Approximately 3 mg of core–shell particles or IC12@AuNP were drop-cast onto the hydrophobic Sigracet 39BB carbon paper. The control blank refers to an untreated carbon paper. The electrochemically active surface area (ECSA) of the catalysts was first assessed *via* double-layer capacitance measurements in 0.5 M KHCO_3_ (Fig. S12 and S13). When comparing the ECSA of OYA@AuNP to that of IC12@AuNP, it was found that NHC functionalisation resulted in a higher ECSA value. Moreover, both core–shell nanoparticles exhibit higher ECSA than OYA@AuNP, as IC12@CSNP, with a lower NHC coverage, has the highest ECSA, followed by IC12@CSNP_NHC_. Linear sweep voltammetry (LSV) measurements were performed between 0 to −1.0 V *vs.* RHE to determine the potential range in which CO_2_RR occurs for our catalysts. As presented in Fig. 4a, the onset of reduction was observed at −0.5 V for IC12@AuNP and reached −15 mA cm^−2^ at −1.0 V. Similar catalytic behaviours were observed for the core–shell nanoparticles, with an onset of reduction at −0.2 V for IC12@CSNP_NHC_ and −0.5 V for IC12@CSNP. The current densities increased to −25 mA cm^−2^ and −17 mA cm^−2^ at −1.0 V, respectively (Fig. 4a).

Chronoamperometry was performed at −0.6, −0.8, and −1.0 V for 15 min under a continuous CO_2_ flow (15 mL min^−1^), with products analysed by online gas chromatography. Only H_2_ and CO were detected as gaseous products, and their faradaic efficiencies (FEs) are shown in Fig. 4b. IC12@AuNP exhibited the highest total FE (%FE_total_), approaching 100% across all applied potentials. In contrast, core–shell systems displayed lower total FEs: 74–88% for IC12@CSNP and 70–78% for IC12@CSNP_NHC_ (Fig. 4b). Post-reaction electrolyte analysis by water-suppressed ^1^H NMR revealed formate as the dominant liquid product for both core–shell catalysts. IC12@CSNP additionally produced methanol, ethanol and acetate, while ethylene glycol and acetate were observed for IC12@CSNP_NHC_ (Fig. S14 and S15). The formation of these liquid products likely accounts for the lower %FE_total_ observed for the core–shell nanoparticles. For IC12@AuNP, the %FE_CO_ was significantly higher than %FE_H_2__, with nearly full CO production capacity at more negative potentials.^8^ In contrast, the bimetallic core–shell catalysts exhibited a shift in selectivity toward H_2_ production. The H_2_ : CO ratio increased with applied potential, from 0.9 : 1 to 2.2 : 1 for IC12@CSNP, and from 2.3 : 1 to 2.7 : 1 for IC12@CSNP_NHC_. ICP-MS analysis confirmed that this behaviour correlates with the Cu–Au composition, indicating that while copper incorporation did not yield appreciable C_2+_ formation, it effectively tuned product selectivity. Similar behaviour has been observed in other CuAu bimetallic catalysts, whereby varying the metal composition alters intermediate adsorption, thereby affecting product selectivity.^22,42^ Furthermore, as Cu is prone to HER, the driving force for H_2_ formation increases with higher Cu content, as observed for IC12@CSNP_NHC_ when compared to IC12@CSNP.

Catalyst stability was assessed by 48 h chronoamperometry in CO_2_-saturated 0.5 M KHCO_3_ at −0.6 V_RHE_, during which both core–shell nanoparticles demonstrated stable current outputs on average of −9.7 mA (IC12@CSNP), and −9.8 mA (IC12@CSNP_NHC_) (Fig. 4c). The formation of H_2_ and CO was monitored over 48 h, revealing that their Faradaic Efficiencies remained stable throughout the period (Fig. S16). The nanoparticles after CO_2_RR were characterised to determine their morphology and oxidation states, thereby assessing particle stability, using STEM, HRTEM, and XPS. The STEM images revealed that the particle sizes after CO_2_RR were 9.1 ± 1, 9.4 ± 1, and 7.2 ± 1 nm for IC12@CSNP, IC12@CSNP_NHC_, and IC12@AuNP, respectively, closely matching the original sizes as above with no visible changes as evidenced by the *d*-spacings characteristics of Cu core and Au shell (Fig. S17 and S18). Furthermore, XPS analysis was conducted using the catalyst-deposited carbon paper, and the N 1s, Au 4f, Cu 2p, and O 1s spectra were evaluated (Fig. S19 and S20). The XPS spectra of IC12@CSNP and IC12@CSNP_NHC_ showed no significant changes when compared to the catalyst before CO_2_RR, whereby the presence of the NHC is still clearly detectable through the characteristic N 1s binding energies at 400.2 eV (IC12@CSNP) and 400.5 eV (IC12@CSNP_NHC_), respectively. These results strongly support the robustness of IC12@CSNP and IC12@CSNP_NHC_ under electrocatalytic conditions.

Although reports on syngas generation with Cu–Au bimetallic nanomaterials are limited, our core–shell systems perform competitively with other reported examples by offering sustained current output (see Table S1 in the SI).^22,43,44^ The structure–activity trends revealed here highlight the dependence of the H_2_ : CO ratio on both the Cu–Au ratio and applied potentials. Given their tunable syngas composition, these electrocatalysts hold promise for applications in methanol, ethanol or Fischer–Tropsch intermediate syntheses.^45^

## Conclusions

In conclusion, we have demonstrated a facile two-step synthesis of NHC functionalised copper–gold core–shell nanoparticles. Our method uses copper nanoparticle seeds onto which a mild bottom-up reduction of gold complexes achieves a distinguishable and air-stable core–shell structure. The IC12 ligand contributes to the stability and dispersity of the particles, as observed by electron microscopy. NHC functionalisation was confirmed to be surface-bound using XPS and Raman spectroscopy measurements. Through the examination of the electrocatalytic activity of IC12@CSNP and IC12@CSNP_NHC_ for CO_2_RR, they exhibited a total faradaic efficiency of 70–88%, and H_2_ : CO ratio ranging between 0.9 : 1 to 2.7 : 1. Interestingly, this ratio can be easily tuned by varying the potential applied. Given the reliable synthetic method and the variation in the H_2_ : CO ratio, these core–shell catalysts are proving themselves to be competent catalysts for electrochemical reactions and syngas production.

## Author contributions

Monnaya Chalermnon: writing – review & editing, investigation (main), formal analysis, data curation, funding acquisition. Robert Richstein: investigation, formal analysis, review & editing. Janine Lichtenberger: investigation, formal analysis, review & editing. Domenico Grammatico: investigation, formal analysis, review & editing. Lingcong Ge: investigation, formal analysis, review & editing. Rachmat Adhi Wibowo: review & editing, supervision, resources, funding acquisition. Jia Min Chin: writing – review & editing, supervision, resources, funding acquisition. Michael R. Reithofer: writing – review & editing, supervision, resources, funding acquisition, conceptualization.

## Conflicts of interest

There are no conflicts to declare.

## Supplementary Material

NR-018-D6NR01169A-s001

## Data Availability

The data supporting this article have been included as part of the supplementary information (SI). Supplementary information is available. See DOI: https://doi.org/10.1039/d6nr01169a.
