# Supplementary material for: Seed-mediated synthesis of NHC-stabilised Cu@Au core–shell nanoparticles from an NHC-Au(i) complex
Source: Nanoscale. 2026 Jun 23;18(29):15697–704. doi: 10.1039/d6nr01169a (PMC13325931; doi:10.1039/d6nr01169a)
Supplement: NR-018-D6NR01169A-s001 [file NR-018-D6NR01169A-s001.pdf]

Supplementary Information (SI) for Nanoscale.
This journal is © The Royal Society of Chemistry 2026

Supplementary Information

# Seed-Mediated Synthesis of NHC-Stabilised Cu@Au Core-Shell Nanoparticles from an NHC-Au(I) Complex

Monnaya Chalermnon,[a] Robert Richstein,[a] Janine Lichtenberger,[b] Domenico Grammatico,[†b] Lingcong Ge,[a] Rachmat Adhi Wibowo,[b] Jia Min Chin,[*c] and Michael R. Reithofer[*a]

[a] Institute of Inorganic Chemistry, Faculty of Chemistry, University of Vienna, Währinger Str. 42, 1090 Vienna, Austria.

[b] Center for Energy, Power and Renewable Gas Systems , AIT Austrian Institute of Technology GmbH, Giefinggasse 2, 1210 Vienna, Austria.

[c] Institute of Functional Materials and Catalysis, Faculty of Chemistry, University of Vienna, Währinger Str. 42, 1090 Vienna, Austria.

[†] Current address: Total Energies One Tech Belgium, Zone Industrielle C Feluy, B-7181 Seneffe, Belgium.

## Chemicals and Instruments

All commercially available chemicals were used without further purification, and all solvents used were purchased from Sigma Aldrich or Acros. Gold(III) Chloride trihydrate ($HAuCl_4.3H_2O$), Oleylamine (80-90%), Oleic acid, Bromotris(triphenylphosphine) copper (II) ($CuBr(PPh_3)_3$) (98%), Imidazole (≥99.5%), Potassium bis(trimethylsilyl)amide (KHMDS) (95%) and tert-butyl amine borane ($tBuNH_3BH_3$; TBAB) were purchased from Sigma Aldrich. 1-Bromododecane ($BrC_{12}H_{25}$) (98%), Potassium carbonate ($K_2CO_3$), Potassium bicarbonate ($KHCO_3$) and sodium hydroxide (NaOH) were purchased from Thermo Fischer.

### *Nuclear Magnetic Resonance (NMR) Spectroscopy*

NMR measurements were conducted with NMR Brucker BioSpin AV Neo 500 MHz or Bruker BioSpin AV III HD 700 MHz. The samples were dissolved in either $CDCl_3$ or DMSO-d6.

### *Mass Spectrometry (MS)*

MS measurements were performed at the Mass Spectrometry Centre, Faculty of Chemistry, University of Vienna, using a Bruker maXis UHR-TOF or Bruker amazon speed ETD.

### *X-ray Photoelectron Spectroscopy (XPS)*

Elemental and oxidation state analysis was performed at the Core Facility "Interface Characterization", using a Nexsa Photoelectron Spectrometer (Thermo Fisher Scientific, UK). The sample was prepared by drop casting of the sample dispersion on a pre-cleaned silicon wafer (~0.25 $cm^2$).

### *Electron Microscopy*

High-Resolution Transmission Electron Microscopy (HRTEM) and High-Angle Annular Dark-Field Scanning Transmission Electron Microscopy (HAADF-STEM) measurements were carried out at the Electron Microscopy Facility at IST Austria using a S/TEM Jeol JEM2800 with the accelerating voltage of 200 kV equipped with a CMOS TEM camera TemCam-XF416, and EDS result was gathered by an EDS detector Jeol Centurio, which is a large solid angle silicon drift detector with 100 $mm^2$ active area for ultrafast elemental mapping of S/TEM samples. The samples were prepared by drop-casting onto copper grids and then dried at 70°C in an oven. Particle size was counted using ImageJ, while the d-spacing and the shell thickness were measured using the EM Measure software package.

### *Inductively Coupled Plasma Mass Spectrometry (ICP-MS)*

ICP-MS was performed using Agilent ICP-MS 7800. Approximately 0.5-1 mg of nanoparticles was digested with aqua regia (3:1 HCl:HNO3), then diluted with Milli-Q water at a 1:10,000 ratio.

*Thermogravimetric Analysis (TGA)*

TGA was conducted using a Mettler-Toledo TGA/DSC 3+ instrument in the temperature range of 25 – 800 °C under $N_2$ atmosphere ($N_2$ flow: 20 mL/min).

*Ultraviolet-visible (UV-Vis) Spectroscopy*

UV-Vis analysis was performed using a PerkinElmer spectrophotometer Lambda 35. The nanoparticles were dispersed in toluene and sonicated for 10 minutes before the measurement. The stability studies were monitored for 24 hours with measurements taken every hour.

*Raman Spectroscopy*

Raman measurements were performed using the confocal Raman microscope (alpha 300 RS; WITec Wissenschaftliche Instrumente und Technologie GmbH, Germany), with a 785 nm laser for IC12Br (power of 39.7 mW), [Au(IC12)Cl] (power of 39.6 mW), IC12@AuNP (power of 0.5 mW), IC12@CSNP (power of 5.0 mW), and IC12@CSNP$_{NHC}$ (power of 1.0 mW). The samples were drop-cast onto a cleaned glass slide or silicon wafer, depending on the suitability. Data analysis was carried out using the WITec Project FIVE software.

**All reactions were carried out under inert conditions using a Mbraun Unilab Pro Glovebox unless otherwise stated.

**Synthesis of 1,3-didodecylimidazolium bromide (IC12Br)**

IC12Br was synthesised according to the modified literature procedure.[1] This reaction was conducted under ambient conditions. In a 250 mL round-bottom flask, imidazole (4 g, 58.8 mmol, 1 equiv.) and $K_2CO_3$ (16.3 g, 117.9 mmol, 2 equiv.) in 80 mL THF were stirred for 1 h at room temperature. Subsequently, $BrC_{12}H_{25}$ (18.2 mL, 75.8 mmol, 1.3 equiv.) was added to the reaction mixture and stirred for 24 hours at room temperature. An additional one equivalent of $BrC_{12}H_{25}$ (18.2 mL, 75.8 mmol, 1.3 equiv.) was then added to the mixture and heated under reflux (80-85°C). The reaction was cooled down to room temperature, and $K_2CO_3$ was removed by filtration. The filtrate was dried *in vacuo* to obtain a viscous yellow liquid. 150 mL of hexane was used to precipitate the viscous liquid into an off-white powder. The final product was filtered and washed with hexane. $^1H$ NMR (500 Mz, $CDCl_3$) δ 10.62 (1H, s, NCHN), 7.32 (2H, t, NCHCHN), 4.34 (4H, t, $NCH_2CH_2$), 1.90 (4H, q, $NCH_2CH_2CH_2$), 1.22 (36H, s, $CH_2$), 0.86 (6H, t, $CH_2CH_3$); $^{13}C$ NMR (126 Mz, $CDCl_3$) δ 137.67, 121.73, 50.27, 32.00, 30.43, 29.70, 29.60, 29.49, 29.43, 29.11, 26.36, 22.78, 14.23. The data is in accordance with the literature.[2]

**Synthesis of chloro[1,3-didodecyl-imidazol-2-ylidene]gold(I) ([Au(IC12)Cl])**

[Au(IC12)Cl] was synthesised according to the modified literature procedure.[3] This reaction was conducted under ambient conditions. In a 10 mL vial, IC12Br (202.9 mg, 0.5 mmol, 1 equiv.) and $Ag_2O$ (57.9 mg, 0.25 mmol, 0.5 equiv.) in 2 mL DCM were stirred for 18 hours at room temperature. The reaction mixture was filtered over Celite into a 20 mL vial to obtain a clear solution. Freshly prepared [Au(THT)Cl] (176.3 mg, 0.55 mmol, 1.1 equiv.) was then added to the filtrate and stirred for 6 h at room temperature. The reaction mixture was once again filtered over Celite, and then the filtrate was dried *in vacuo* to yield a yellow oil. The crude material was purified using column chromatography with a 1:1 (vol%) hexane:ethyl acetate mixture. Finally, the crude was precipitated in pentane to obtain a white powder. $^1H$ NMR (700 Mz, $CDCl_3$) δ 6,92 (2H, s, NCHCHN), 4.14 (4H, t, $NCH_2CH_2$), 1.83 (4H, q, $NCH_2CH_2CH_2$), 1.25 (36H, s, $CH_2$), 0.88 (6H, t, $CH_2CH_3$); $^{13}C$ NMR (176 Mz, $CDCl_3$) δ 170.54, 120.36, 51.71, 32.05, 31.12, 29.75, 29.66, 29.58, 29.48, 29.28, 26.57, 22.83, 14.26. The data is in accordance with the literature.[4]

**Synthesis of IC12-functionalised copper-gold core-shell nanoparticles (IC12@CSNP)**

The synthesis of core-shell nanoparticles was divided into two parts.[5] OYA-OA@CuNP was synthesised according to the modified literature procedure [6]. In a 20 mL vial, $CuBr(PPh_3)_3$ (94.4 mg, 0.1 mmol, 1 equiv.) and oleylamine (208 µL, 0.63 mmol, 6.3 equiv.) in 7 mL toluene were stirred at 80°C for 10 minutes. *tert*-butylamine borane (TBAB) (86.6 mg, 1 mmol, 10 equiv.) was added rapidly into the mixture and stirred for 1 hour at 80°C. A colour change was observed from colourless to deep red. The reaction mixture was removed from the heat and cooled down to room temperature while stirring. Oleic acid (160 µL, 0.51 mmol, 5.1 equiv.) was added to the deep red solution and stirred for another 30 minutes. The synthesised OYA-OA@CuNP in toluene was purified by centrifugation at 13,000 rpm for 15 minutes. After every two centrifugations, the red-black precipitate was collected into a pre-weighted vial. The centrifugation step was repeated five times. The collected solid was dried *in vacuo* for 18-24 h. The dried solid was weighed and

redispersed in toluene to create a concentration of 10 mg/mL. The redispersed OYA-OA@CuNP had a deep red colour. No characterisation was done at this step.

The synthesis of IC12@CSNP was as follows. In a 10 mL vial containing the OYA-OA@CuNP solution in toluene (concentration: 10 mg/mL), [Au(IC12)Cl] (Cu:Au 5.5 equiv.) was added into the solution and stirred for 10 minutes at 50°C. In a separate vial, TBAB (1.6 equiv. to the [Au(IC12)Cl]) was dissolved in toluene at a concentration of 10 mg/mL and also heated for 10 minutes at 50°C to obtain a clear solution. Once both solutions were at the same temperature, the TBAB solution was added rapidly in a single portion to the copper-gold mixture. The reaction mixture was stirred for 6 hours at 50°C to obtain a deep red suspension. The suspension was removed from the glove box for the subsequent purification steps. A few drops of MilliQ water were added to quench the reaction. The crude was then added dropwise into 60 mL of acetone to precipitate. The samples were centrifuged at 8,000 rpm for 8 minutes, then washed once with acetone and twice with a 1:1 (vol%) mixture of acetone/ethyl acetate. The nanoparticles were redispersed in toluene.

The same preparation method was adapted for $IC12@CSNP_{NHC}$, but four equivalents of free carbene (deprotonated IC12Br) to [Au(IC12)Cl] were added in addition to the gold complex. The free carbene of IC12Br was synthesised by reacting IC12Br (1.1 equiv.) with KHMDS (1 equiv.) in 1 mL toluene. The reaction was stirred for 1.5 h at room temperature, then the crude was filtered through a 0.22 µm filter. The collected filtrate was combined with the copper-gold mixture and stirred for 10 minutes at 50°C. In a separate vial, TBAB (1.6 equiv.) was dissolved in toluene at a concentration of 10 mg/mL and also heated for 10 minutes at 50°C. Once both solutions were at the same temperature, the TBAB solution was added rapidly in a single portion to the mixture. The reaction mixture was stirred for 6 hours at 50°C to obtain a deep red suspension. The purification step was similar to IC12@CSNP.

**Synthesis of IC12-functionalised gold nanoparticles (IC12@AuNP)**

IC12@AuNP was synthesised according to the modified literature procedure.[7] In a 10 mL vial, [Au(IC12)Cl] (34 mg, 0.2 mmol, 1 equiv.) was dissolved in 1 mL of toluene and stirred at room temperature until a homogeneous solution was obtained. In a separate vial, $tBuNH_2BH_3$ (87 mg, 1.0 mmol, 5 equiv.) was dissolved in toluene at a concentration of 50 mg/mL and also stirred at room temperature until homogeneous. The $tBuNH_2BH_3$ solution was added rapidly in a single portion to the gold solution. The reaction mixture was stirred for 2 hours at room temperature. A few drops of MilliQ water were added to quench the reaction. The same purification steps as IC12@CSNP were carried out. Finally, the nanoparticles were redispersed in toluene.

**Synthesis of oleylamine-functionalised gold nanoparticles (OYA@AuNP)**

OYA@AuNP were synthesised according to the literature procedure.[8] In a 10 mL vial, $HAuCl_4.3H_2O$ (119 mg, 0.3 mmol, 1 equiv.) and oleylamine (2 mL, 6.1 mmol, 20 equiv.) were mixed and sonicated until homogeneous. In a three-neck flask purged with argon, oleylamine (4 mL, 12.1 mmol, 40 equiv.) was heated to 150°C, and then the previous gold solution was rapidly injected in a single portion. The reaction mixture was stirred at 150°C for 40 minutes. The crude was a red solution, which was added dropwise into 60 mL of acetone. Then, it was centrifuged at 8,000 rpm for 8 minutes, washed once with acetone, and twice with ethyl acetate. Finally, the nanoparticles were redispersed in toluene.

## Electrochemical Evaluation

The working electrode was prepared *via* the drop-casting method. The catalyst was loaded onto the end of the carbon paper (2 x 1 cm; h x w), with the coverage area of the nanoparticles limited to 1 $cm^2$ to leave the remaining space available for attachment to the holder. The synthesised nanoparticles were redispersed in toluene at a concentration of 10 mg/mL, then drop-cast and dried until approximately 3 mg of the catalyst was on the ®SIGRACET 39BB carbon paper (SGL Carbon, Fuelcell Store). After casting, the electrode was further dried for 30 minutes in an oven at 70°C. All electrodes were freshly prepared before each electrochemical experiment.

The ECSA was evaluated using the double-layer capacitance measurement.[9, 10] In a H-cell, both cathodic and anodic chambers were filled with 30 mL of Ar-saturated 0.5 M $KHCO_3$. Ag/AgCl (3M KCl) and Pt mesh were used as the reference electrode and counter electrode, respectively. The catalyst was activated by scanning cyclic voltammetry curves between -2 to 2 V. The non-faradaic region was determined with the CV, and the selected potential range was scanned at various scan rates (10, 20, 40, 60, 80, 100 and 120 mV/s). The difference in the current is plotted against the scan rate to obtain the slope ($C_{dl}$). The specific capacitance is assumed to be 0.04 mF/$cm^2$.[11, 12] The ECSA are normalised against the weight of the nanoparticles cast onto the carbon paper.

$$ECSA = C_{dl}/C_s$$

$$C_{dl} = \Delta J/2 = (J_a - J_c)/2$$

The electrochemical $CO_2$ reduction experiments were carried out in a customised air-tight H-cell separated by a Fumasep bipolar membrane (FuMA-Tech). Ag/AgCl (3M KCl) and Pt wire were used as the reference electrode and counter electrode, respectively. The working compartment held 20 mL of 0.5 M $KHCO_3$. Prior to every measurement, the electrolyte was purged with $CO_2$ for at least 15 minutes at a flow rate of 15 mL/min (pH 7.2) and only the catalyst section of the working electrode was immersed in the electrolyte solution. Linear sweep voltammetry (LSV) was conducted at a scan rate of 50 mV/s within an applied potential range of 0 to -1.6 V (vs Ag/AgCl). To determine the $CO_2$ reduction activity, chronoamperometry experiments were conducted at three selected potentials of -1.2 V, -1.4 V and -1.6 V (vs. Ag/AgCl) for 15 minutes. During these experiments, $CO_2$ was continuously passed through the cathodic compartment of the H-cell at a rate of 15 ml/min. The gaseous products were analysed online using gas chromatography (Shimadzu GC-2030) equipped with a ShinCarbon ST Micropacked column and a Dielectric-Barrier Discharge Ionisation Detector (BID). The measurements were performed in triplicates and averaged to obtain the final data.

All the potential recorded are converted to RHE using the equation:[11]

$$E(vs.\ RHE) = E(vs.\ Ag/AgCl) + 0.197\ V + (0.059 \times pH)$$

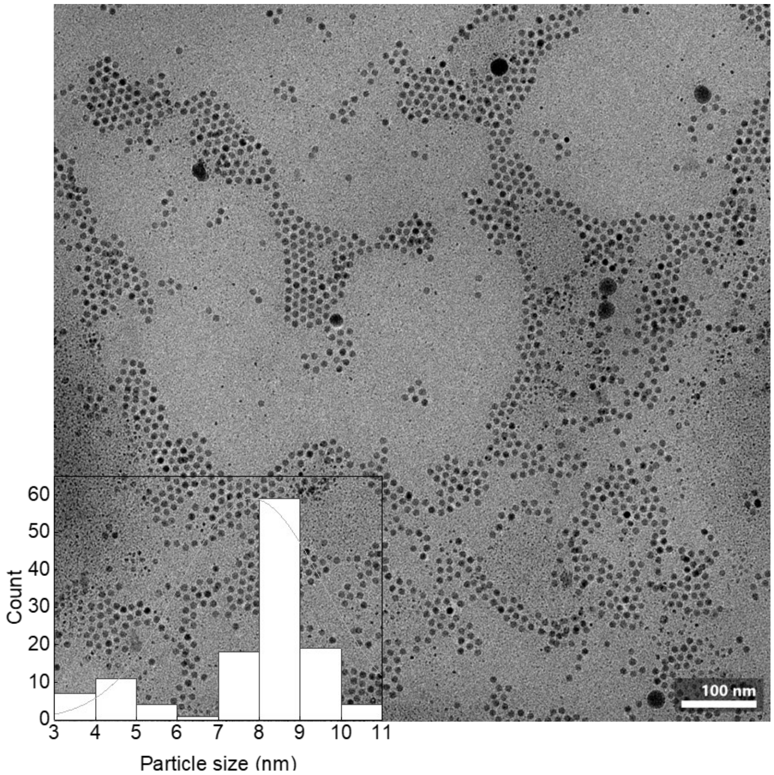


**Fig. S1**: TEM of OYA-OA@CuNP (Particles size: 7.90 ± 2 nm)

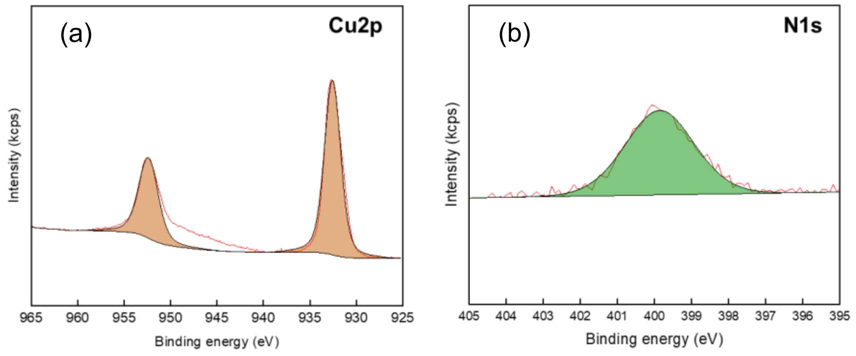


**Fig. S2**: XPS spectra of OYA-OA@CuNP (a) Cu2p and (b) N1s.

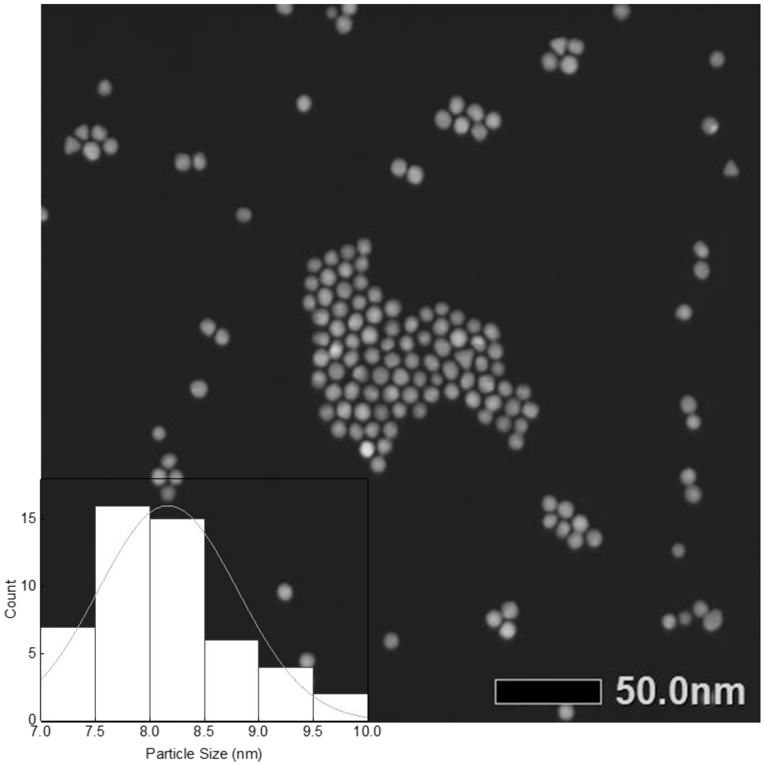


**Fig. S3**: STEM of IC12@AuNP

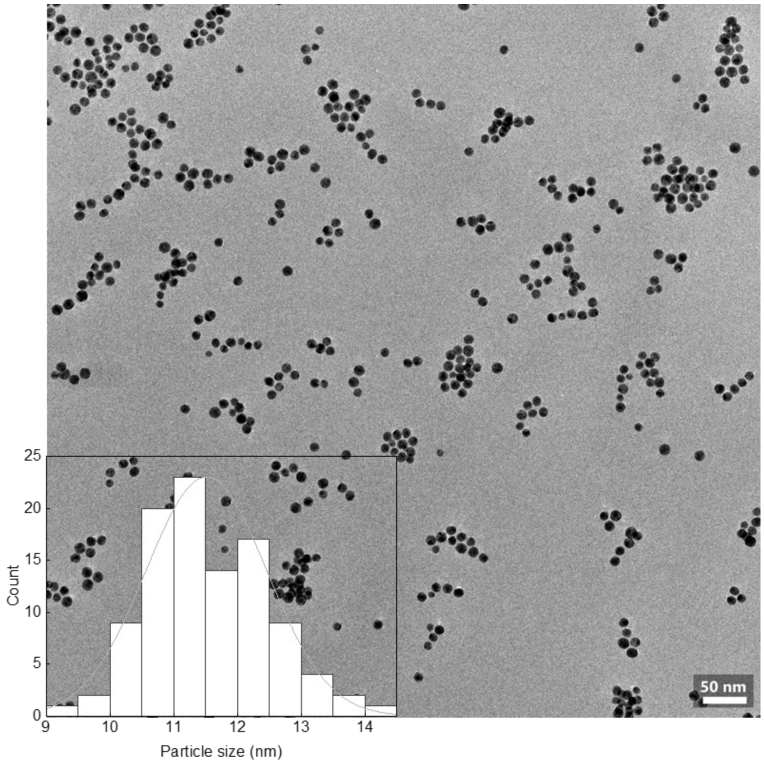


**Fig. S4**: TEM of OYA@AuNP

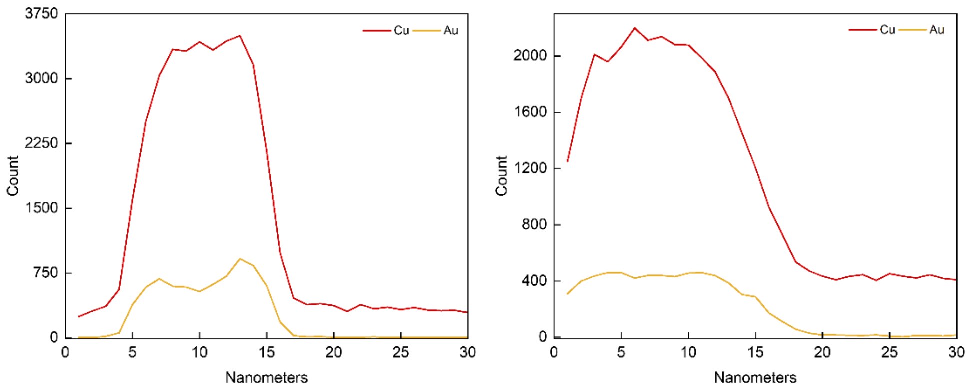


**Fig. S5**: EDS line scan of (a) IC12@CSNP and (b) $IC12@CSNP_{NHC}$

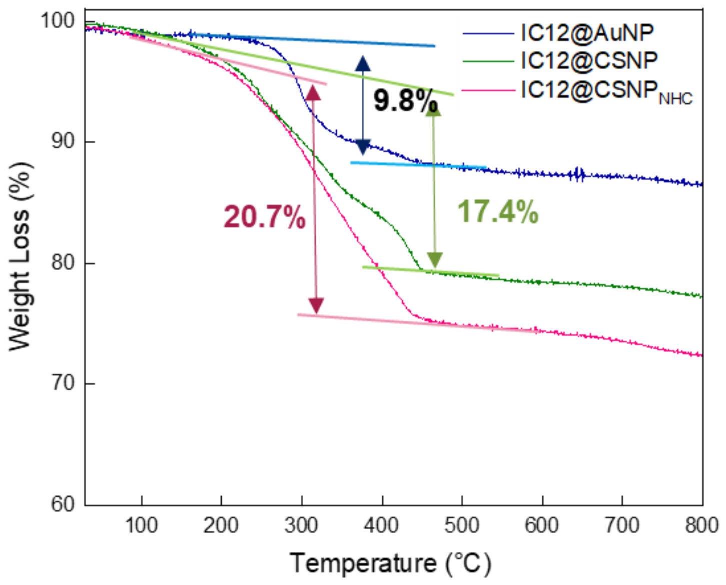


**Fig. S6**: TGA of IC12@AuNP, IC12@CSNP and $IC12@CSNP_{NHC}$

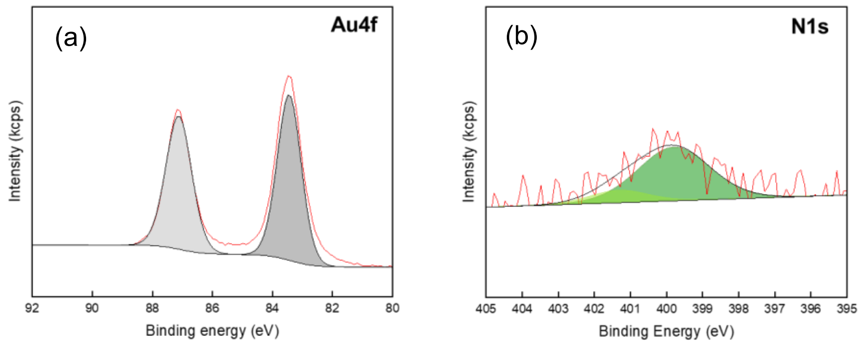


**Fig. S7**: XPS spectra of IC12@AuNP (a) Au4f and (b) N1s.

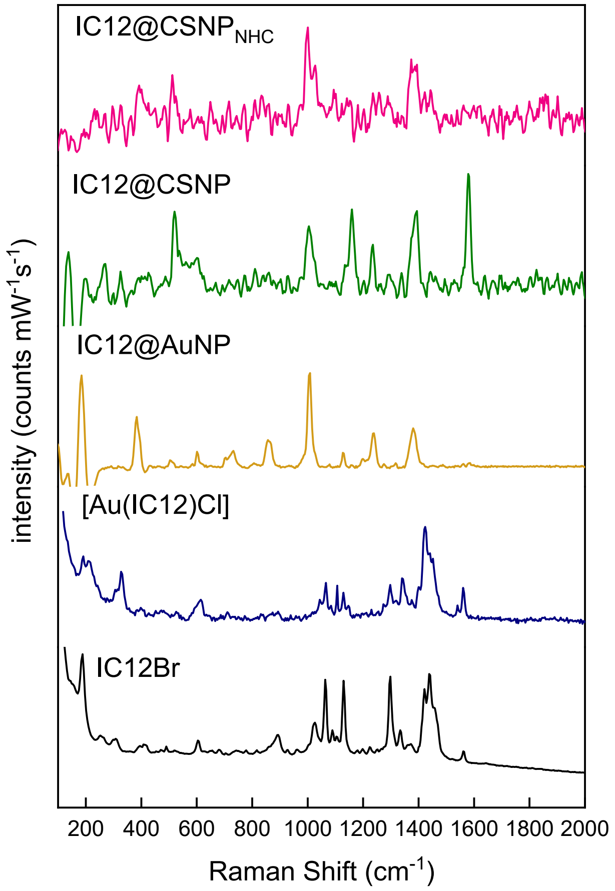


**Fig. S8**: Raman spectra of IC12Br, [AuCl(IC12)], IC12@AuNP, IC12@CSNP, and IC12@CSNP$_{NHC}$

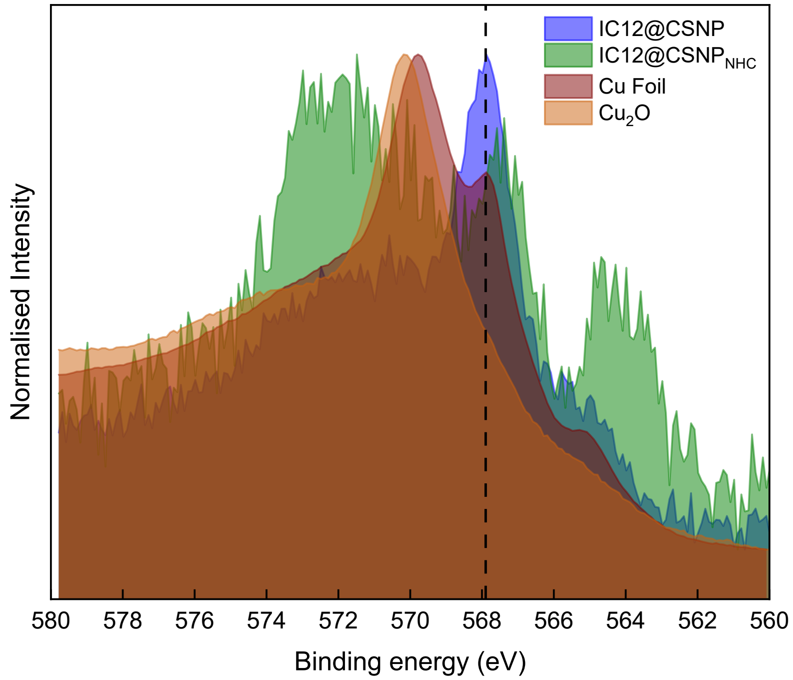


**Fig. S9**: Auger spectra of IC12@CSNP, IC12@CSNP$_{NHC}$, Cu Foil, and $Cu_2O$

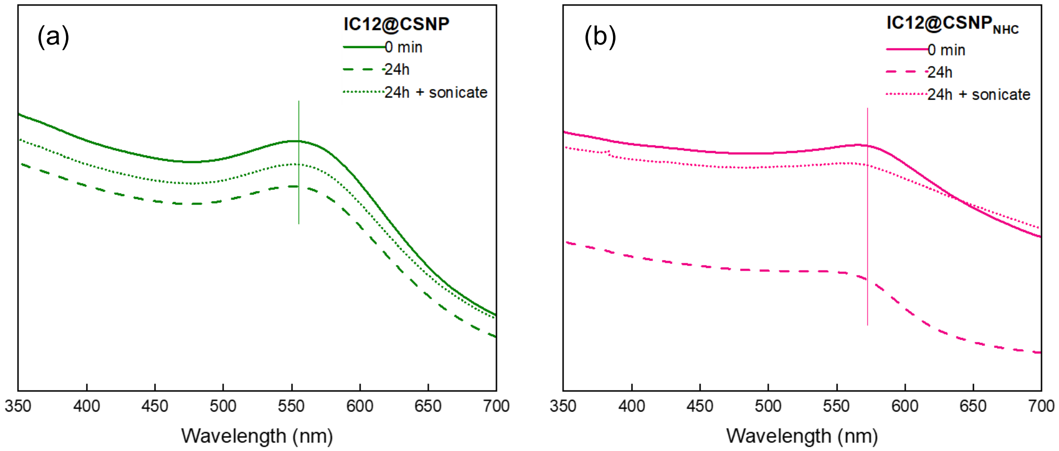


**Fig. S10**: UV-Vis spectra of (a) IC12@CSNP and (b) IC12@CSNP$_{NHC}$; solid line = measured at 0 min, dash line = after 24 hours, and dotted line = after 24 hours and sonicated

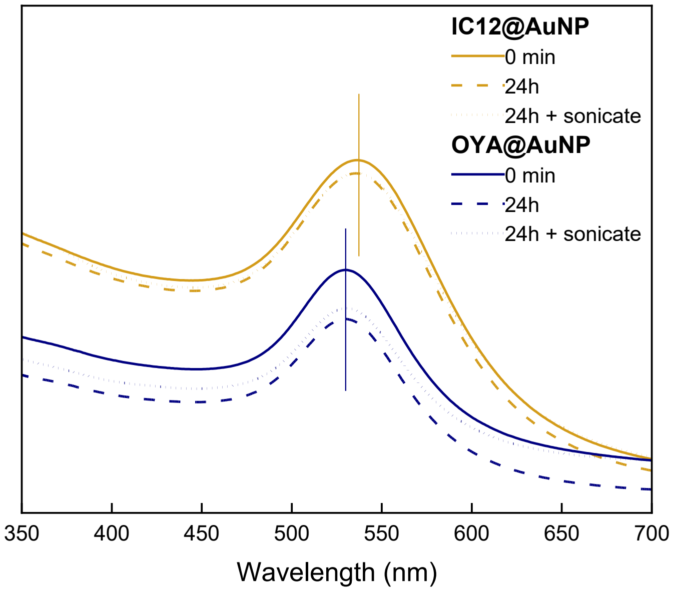


**Fig. S11**: UV-Vis spectra of IC12@AuNP and OYA@AuNP; solid line = measured at 0 min, dash line = after 24 hours, and dotted line = after 24 hours and sonicated

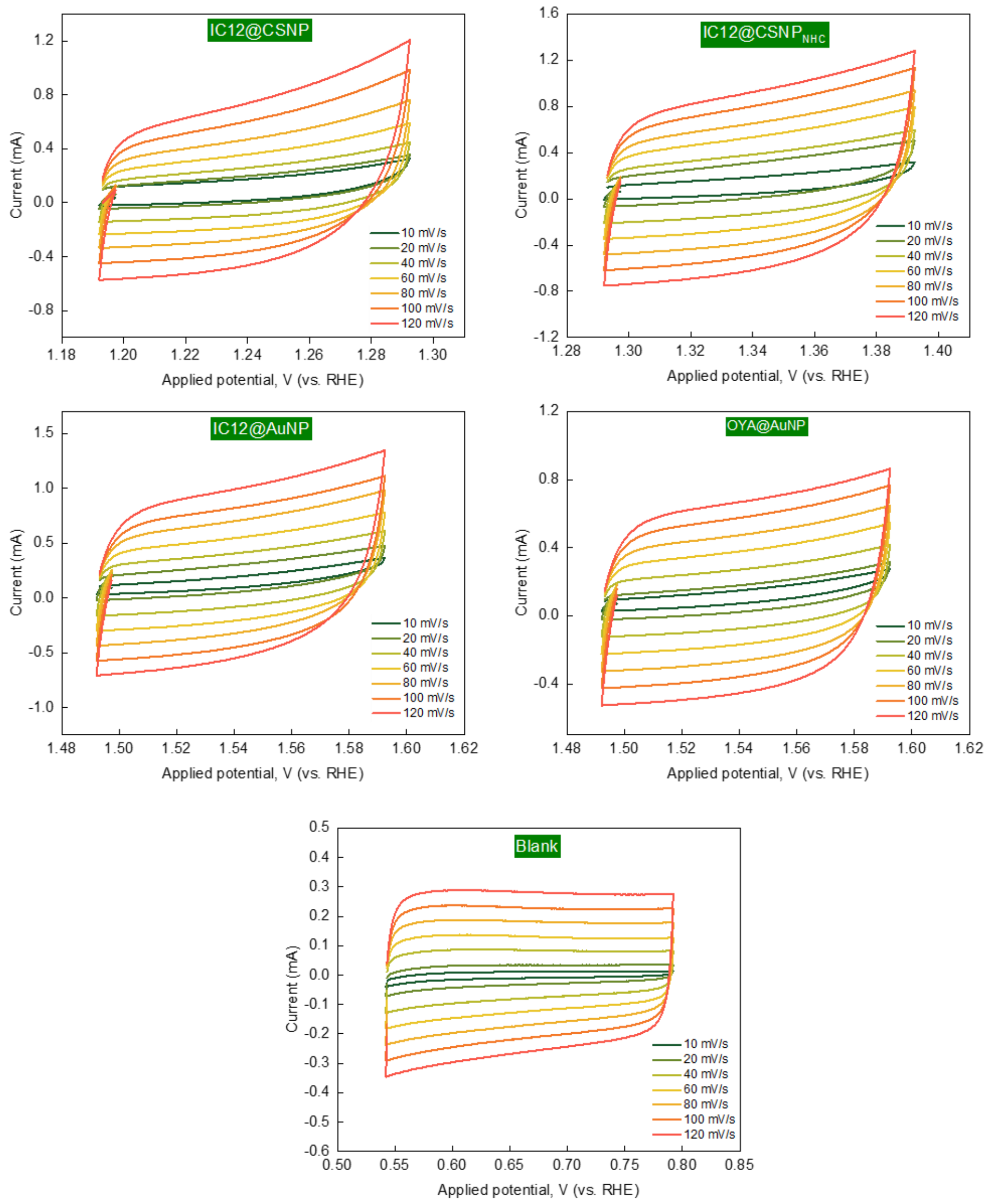


**Fig. S12**: Cyclic voltammetry (CV) curves of IC12@CSNP, IC12@CSNP$_{NHC}$, IC12@AuNP, OYA@AuNP and Blank

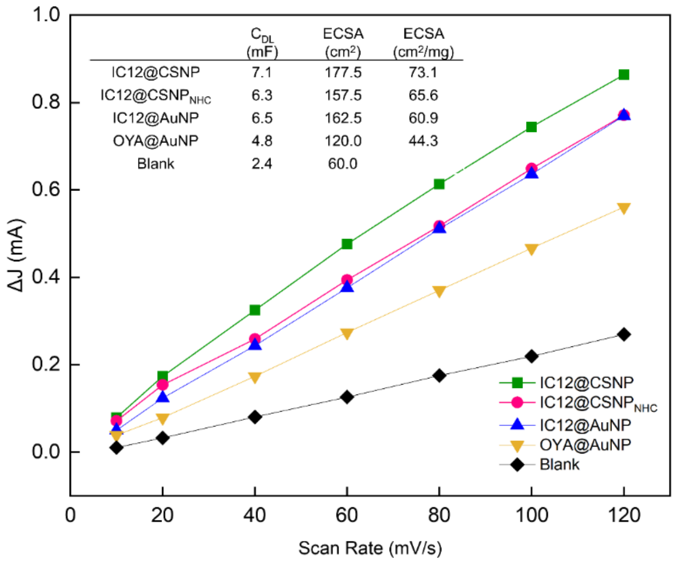


**Fig. S13**: Electrochemical double-layer plot and ECSA values for IC12@CSNP, IC12@CSNP$_{NHC}$, IC12@AuNP, OYA@AuNP, and blank.

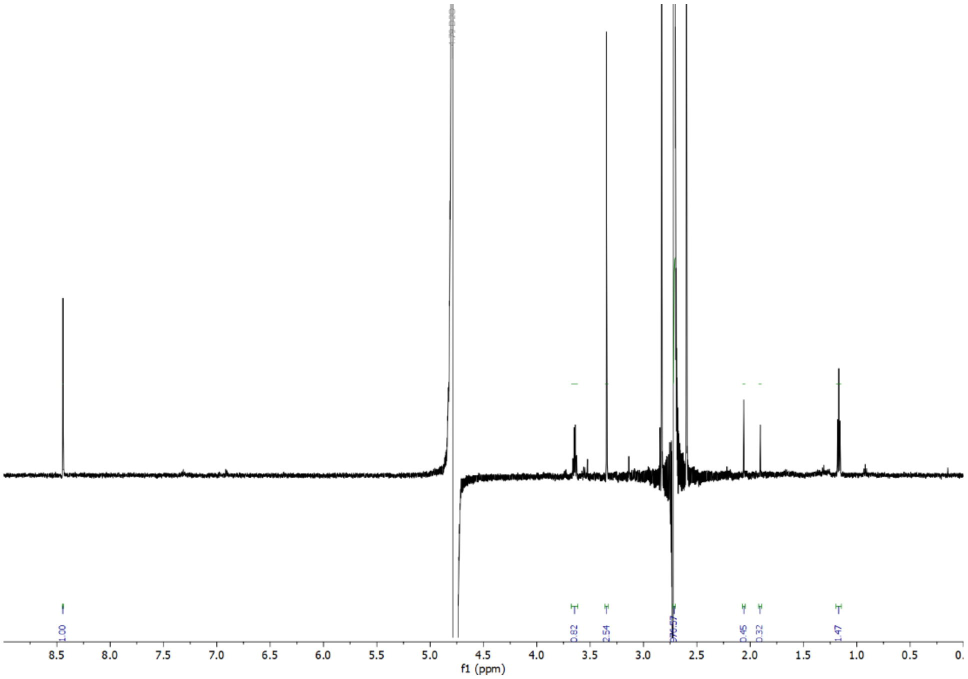


**Fig. S14**: Water-suppressed $^{1}$H NMR of IC12@CSNP from the chronoamperometry measurement.

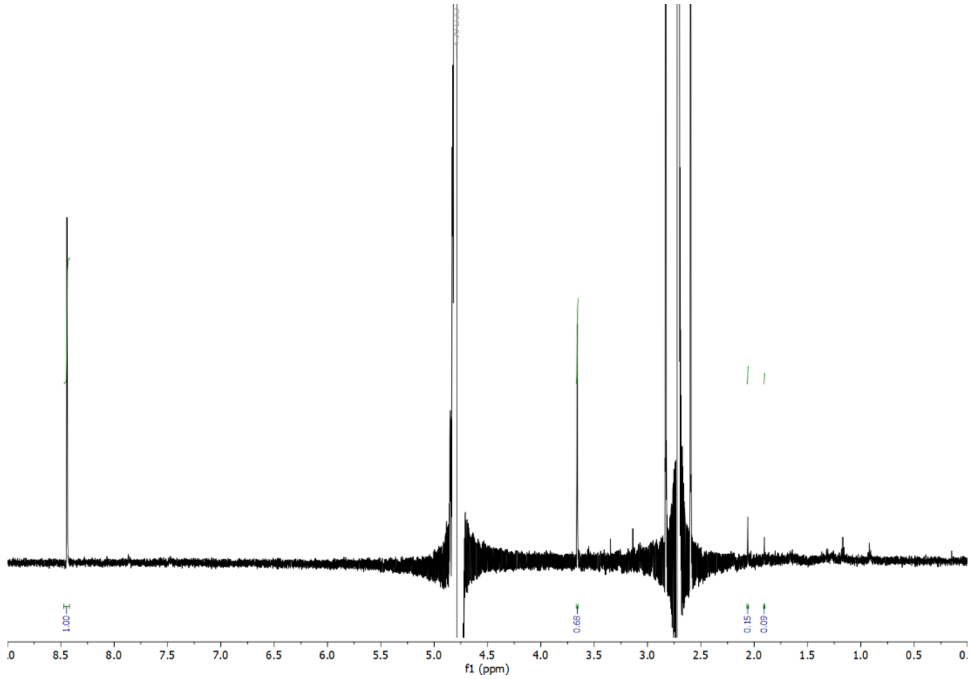


**Fig. S15**: Water-suppressed $^{1}$H NMR of IC12@CSNP$_{NHC}$ from the chronoamperometry measurement.

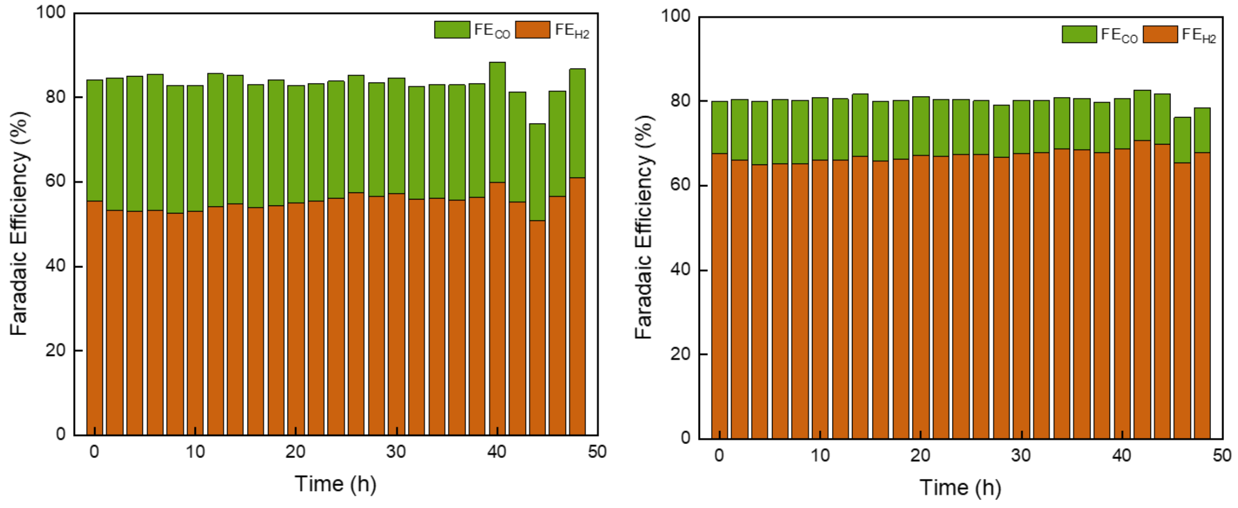


**Fig. S16**: Faradaic efficiencies of $H_2$ and CO monitored by GC during the 48-hour stability test of (a) IC12@CSNP and (b) IC12@CSNP$_{NHC}$

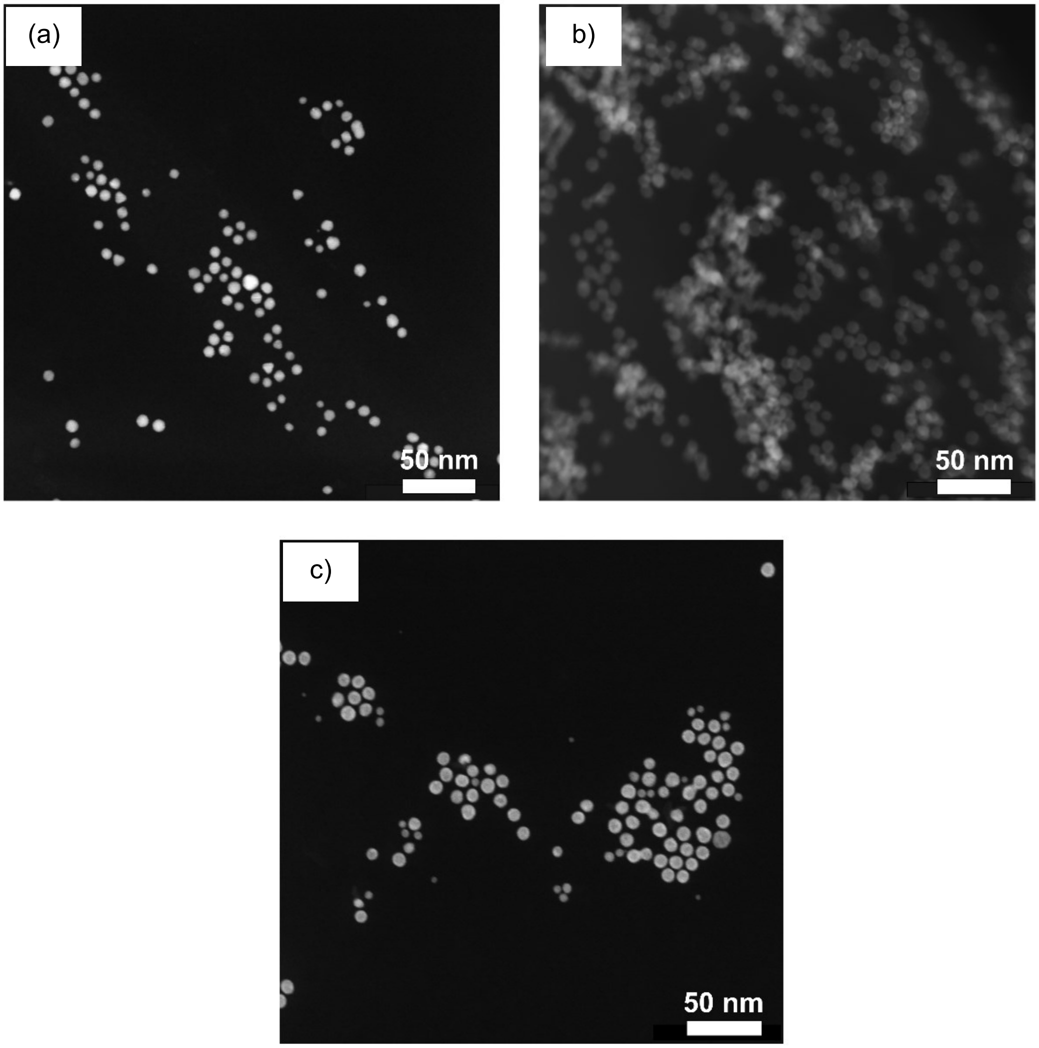


**Fig. S17**: STEM images of nanoparticles after the $CO_2$RR experiment. (a) IC12@AuNP, (b) IC12@CSNP, and (c) IC12@CSNP$_{NHC}$. The nanoparticles were removed from the carbon paper for STEM and HRTEM measurements by sonicating the electrode in toluene.

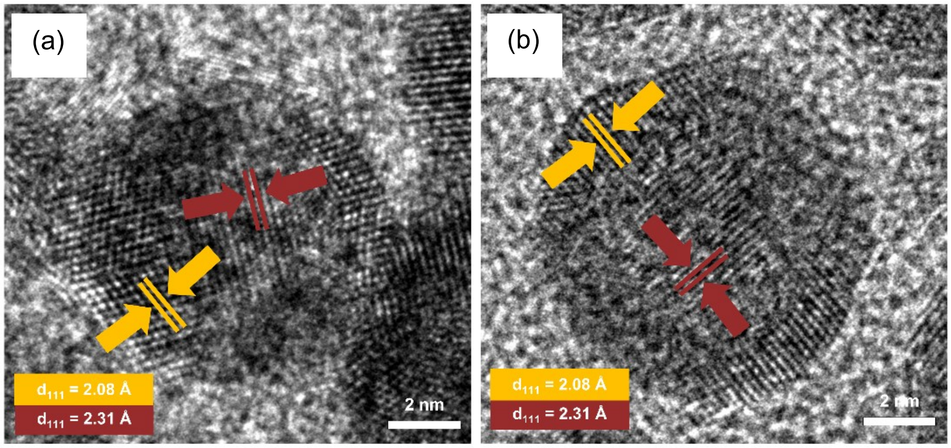


**Fig. S18**: HRTEM images of nanoparticles after the $CO_2$RR experiment. (a) IC12@CSNP, and (b) IC12@CSNP$_{NHC}$

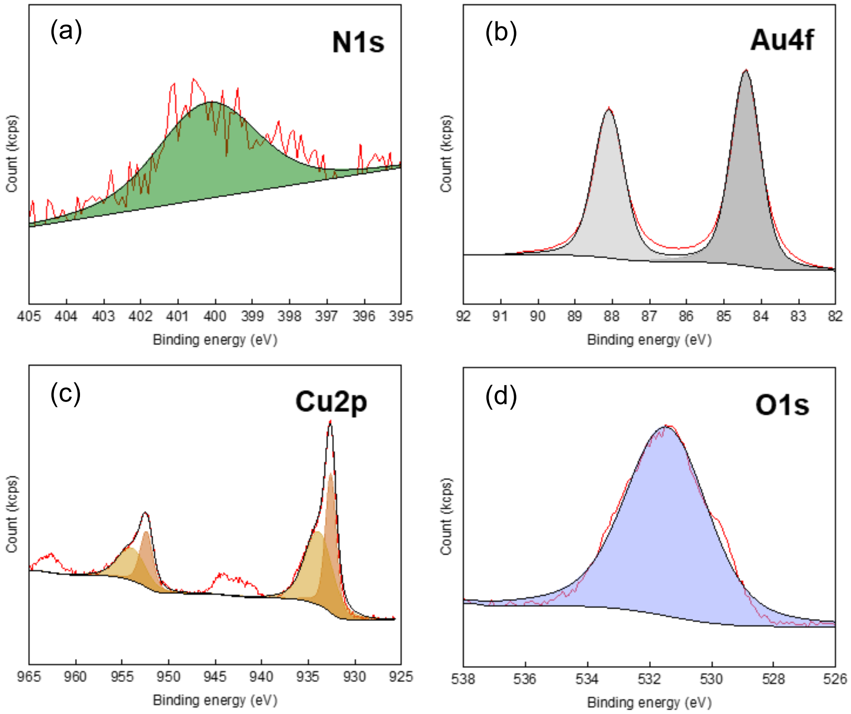


**Fig. S19**: XPS spectra of IC12@CSNP: a) N1s, b) Au4f, c) Cu2p, and d) O1s

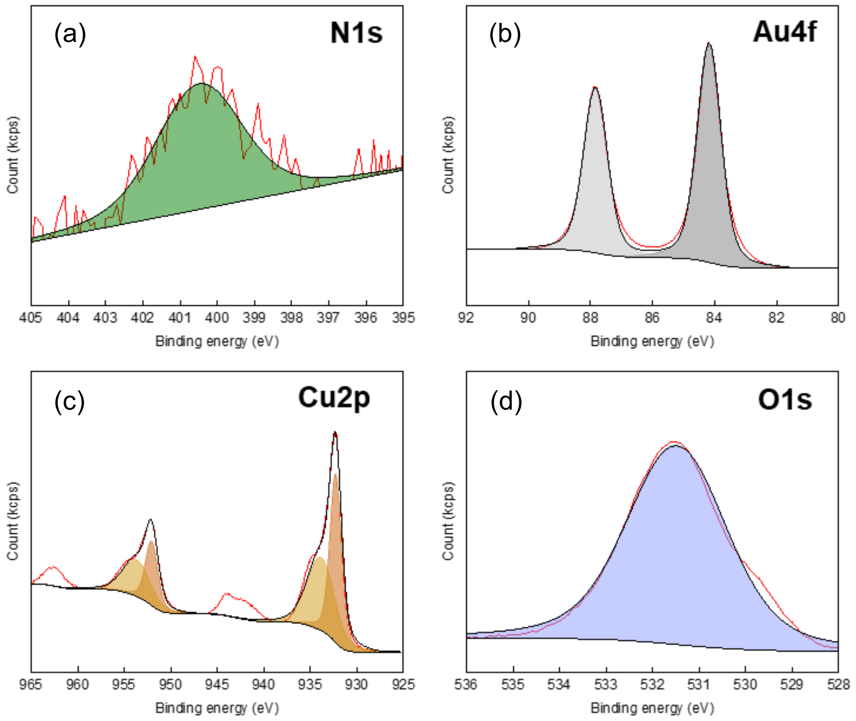


**Fig. S20**: XPS spectra of IC12@CSNP$_{NHC}$: a) N1s, b) Au4f, c) Cu2p, and d) O1s

**Table S1**: Summary of reported work on syngas production based on copper-gold bimetallic nanoparticles

| Material | Potential range (vs RHE) | Electrolyte | Syngas $H_2$:CO ratio | Stability | Refs |
|---|---|---|---|---|---|
| o-AuCu | -0.4 to -1.1 V | 0.1 M $KHCO_3$ | 6:1 to 1:6 | ~ -7.5 mA cm$^{-2}$ at –0.9 V (vs. RHE) over 4 h | 13 |
| $AuCu_3$-100<br>$AuCu_3$-200<br>$AuCu_3$-300 | -0.5 to - 1.0 V | 0.1 M $KHCO_3$ | 5:1 to 1:6<br>2:1 to 1:1.2<br>7:1 | ~ -40 mA cm$^{-2}$ at –1.0 V (vs. RHE), over 10 h | 14 |
| $AuCu_2$/CNT | -0.3 to -1.0 V | 0.5 M $KHCO_3$ | 0.12 to 5.24 | ~ -1.3 mA cm$^{-2}$ at –0.4 V (vs. RHE), over 20 h | 15 |
| Au-coated Cu nanowire electrode | -0.65 V | 0.5 M $KHCO_3$ | 2:1 | ~ -8 mA cm$^{-2}$ at –0.65 V (vs. RHE), over 24 h | 16 |
| IC12@CSNP<br>IC12@CSNP$_{NHC}$ | -0.6 to -1.0 V | 0.5 M $KHCO_3$ | 0.5:1 to 1.7:1<br>1.6:1 to 3.5:1 | ~ -9.7 mA<br>~ -9.8 mA<br>at –0.6 V (vs. RHE) over 48 h | This work |

**ADDITIONAL SUPPLEMENTARY INFORMATION**

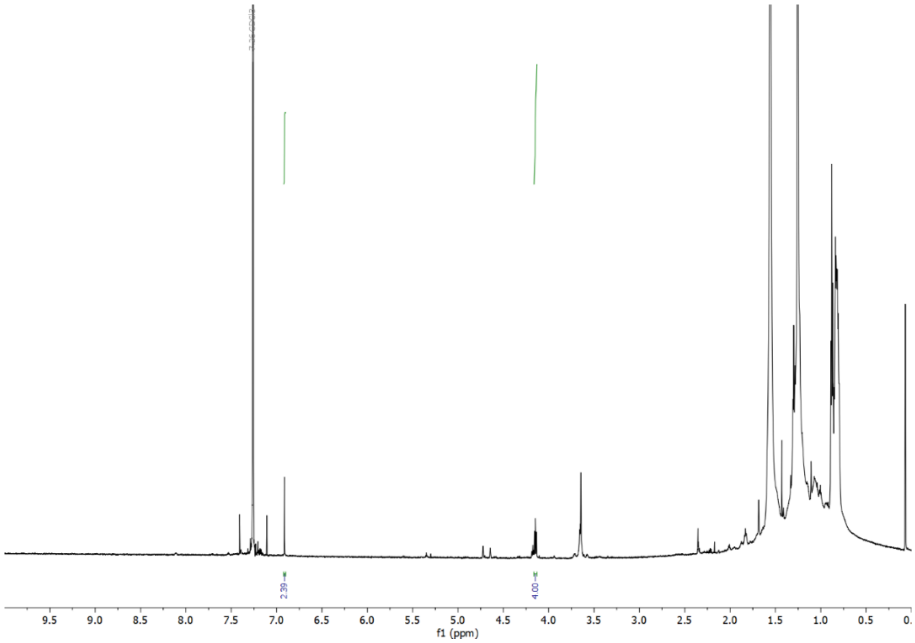


**Fig. S21**: $^{1}$H NMR of IC12@AuNP

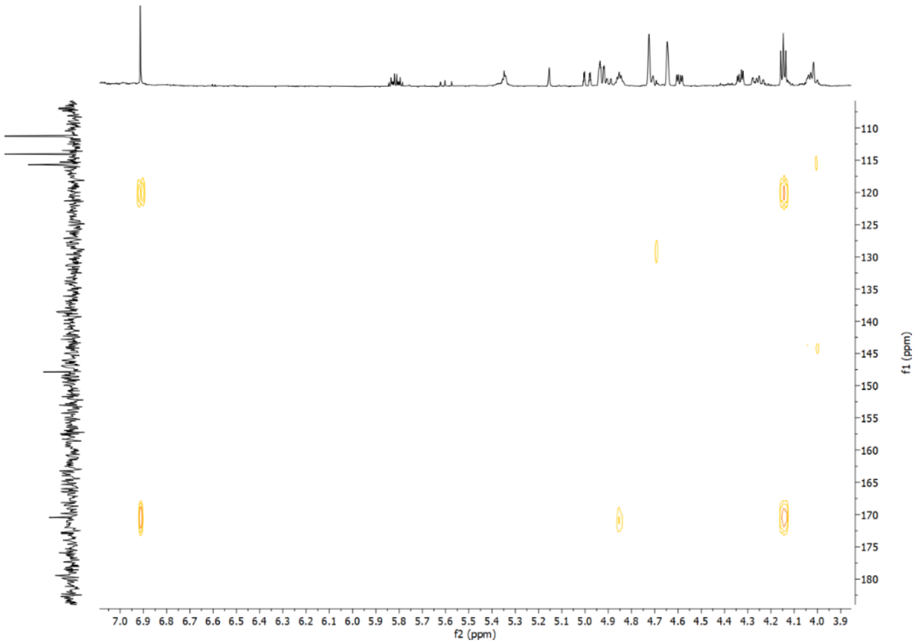


**Fig. S22**: HMBC of IC12@AuNP

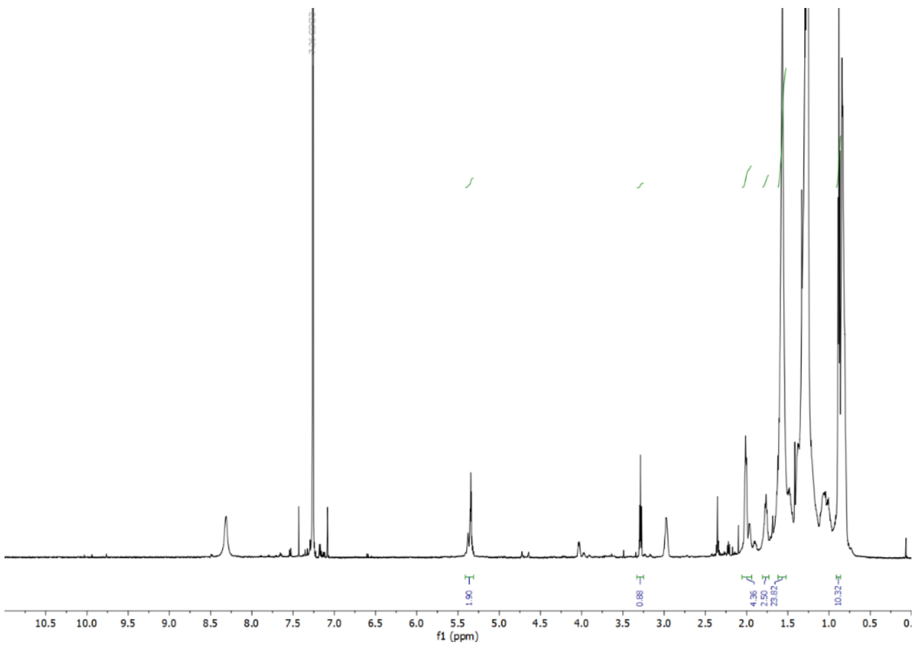

**Fig. S23**: $^1$H NMR of OYA@AuNP

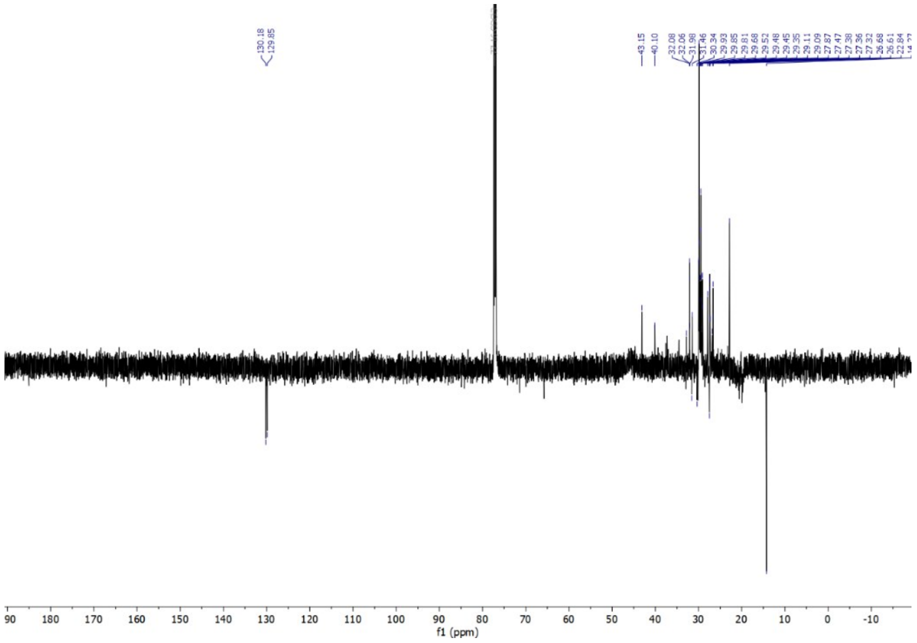

**Fig. S24**: $^{13}$C NMR of OYA@AuNP

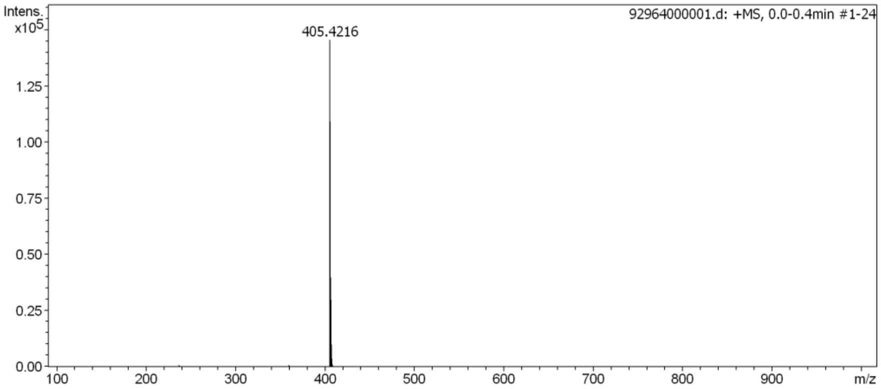


**Fig. S25**: Mass spectra of IC12Br

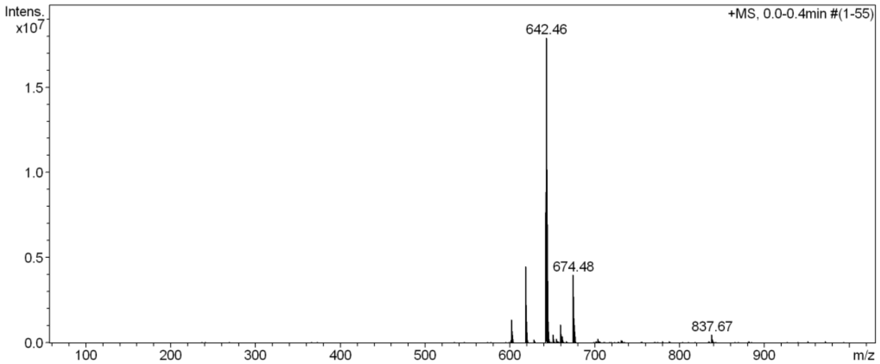


**Fig. S26**: Mass spectra of [Au(IC12)Cl]

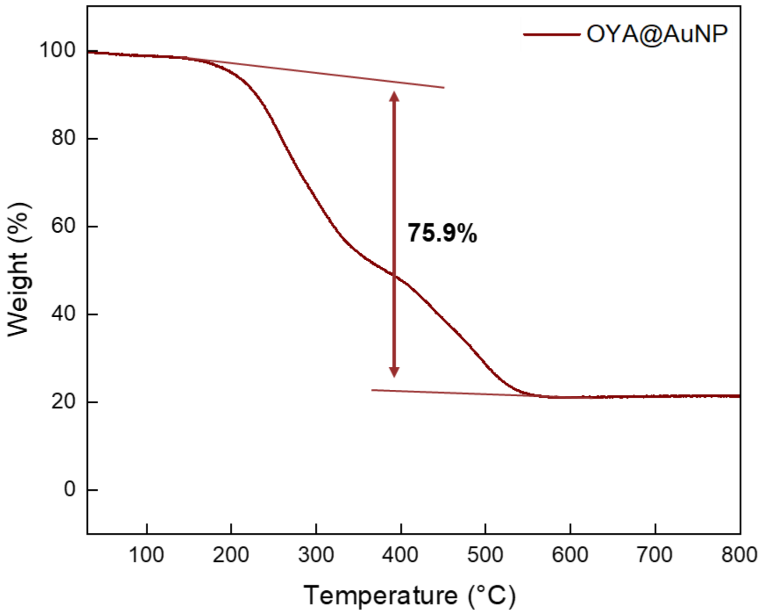


**Fig. S27**: TGA of OYA@AuNP

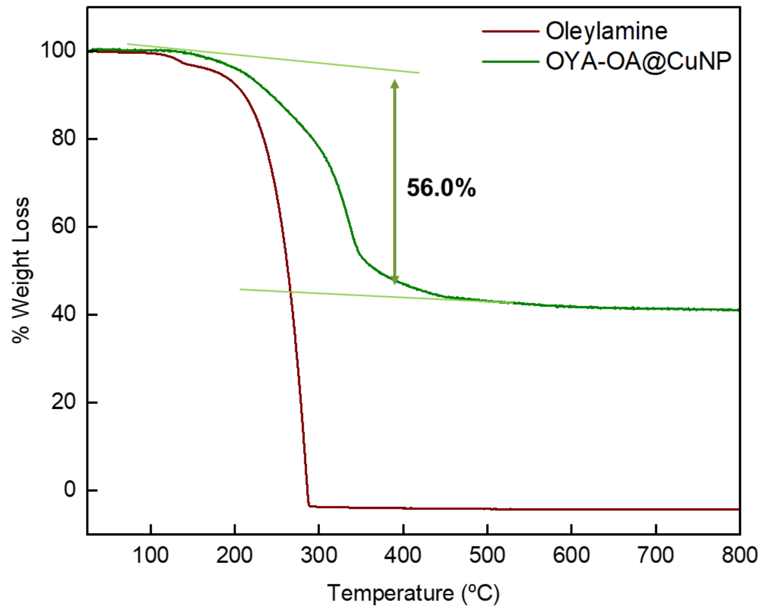


**Fig. S28**: TGA of OYA-OA@CuNP

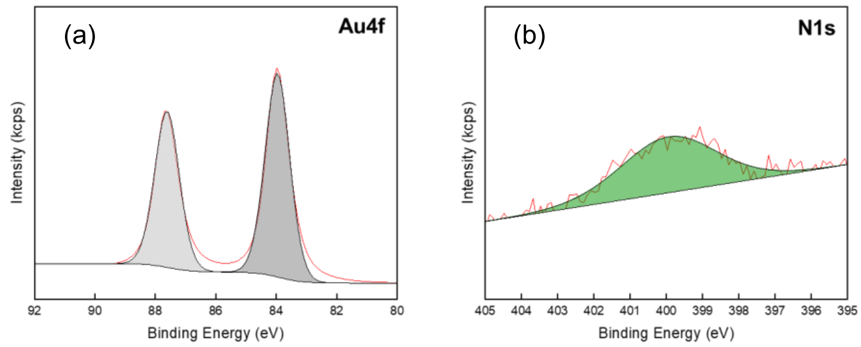


**Fig. S29**: XPS spectra of OYA@AuNP: (a) Au4f and (b) N1s

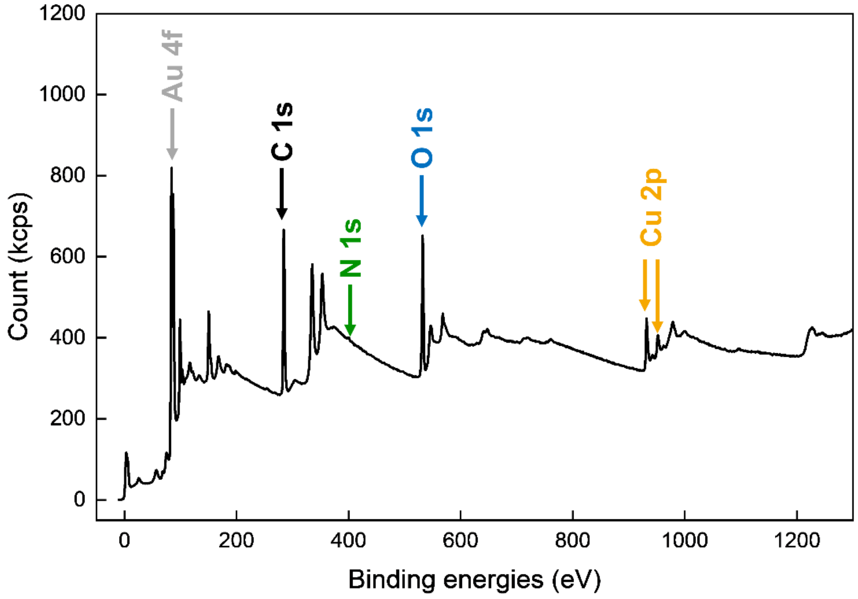


**Fig. S30**: Survey XPS of IC12@CSNP

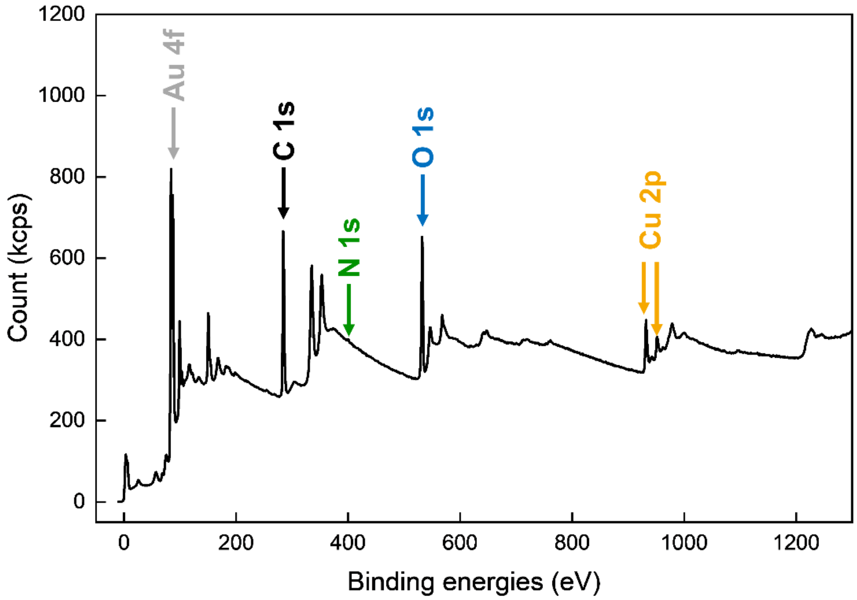


**Fig. S31**: Survey XPS of IC12@CSNP$_{NHC}$

## References

1. X. Frogneux, L. Hippolyte, D. Mercier, D. Portehault, C. Chanéac, C. Sanchez, P. Marcus, F. Ribot, L. Fensterbank and S. Carenco, Chemistry – A European Journal, 2019, 25, 11481-11485.
2. N. Bridonneau, L. Hippolyte, D. Mercier, D. Portehault, M. Desage-El Murr, P. Marcus, L. Fensterbank, C. Chanéac and F. Ribot, Dalton Transactions, 2018, 47, 6850-6859.
3. P. de Frémont, N. M. Scott, E. D. Stevens and S. P. Nolan, Organometallics, 2005, 24, 2411-2418.
4. R. T. W. Huang, W. C. Wang, R. Y. Yang, J. T. Lu and I. J. B. Lin, Dalton Transactions, 2009, https://doi.org/10.1039/b907171g7121.
5. E. Larios, Z. Molina, A. Maldonado and J. Tanori, Journal of Dispersion Science and Technology, 2012, 33, 719-723.
6. M. A. Ben Aissa, B. Tremblay, A. Andrieux-Ledier, E. Maisonhaute, N. Raouafi and A. Courty, Nanoscale, 2015, 7, 3189-3195.
7. G. M. D. M. Rúbio, B. K. Keppler, J. M. Chin and M. R. Reithofer, Chemistry – A European Journal, 2020, 26, 15859-15862.
8. N. A. Nosratabad, Z. Jin, L. Du, M. Thakur and H. Mattoussi, Chemistry of Materials, 2021, 33, 921-933.
9. J. Huang, J. Dai, J. Zhu, R. Chen, X. Fu, H. Liu and G. Li, Journal of Catalysis, 2022, 415, 134-141.

10. Z. Cao, D. Kim, D. Hong, Y. Yu, J. Xu, S. Lin, X. Wen, E. M. Nichols, K. Jeong, J. A. Reimer, P. Yang and C. J. Chang, Journal of the American Chemical Society, 2016, 138, 8120-8125.
11. X. Ma, Y. Shen, S. Yao, C. An, W. Zhang, J. Zhu, R. Si, C. Guo and C. An, Journal of Materials Chemistry A, 2020, 8, 3344-3350.
12. J. Hao, Z. Zhuang, K. Cao, G. Gao, C. Wang, F. Lai, S. Lu, P. Ma, W. Dong, T. Liu, M. Du and H. Zhu, Nature Communications, 2022, 13, 2662.
13. Y. Han, Z. Wang, X. Han, W. Fang, Y. Zhou, K. Lei, B. You, H. S. Park and B. Y. Xia, ACS Sustainable Chemistry & Engineering, 2021, 9, 2609-2615.
14. C. An, Y. Shen, W. Yan, L. Dai and C. An, Nano Research, 2021, 14, 3907-3912.
15. H. Chen, Z. Li, Z. Zhang, K. Jie, J. Li, H. Li, S. Mao, D. Wang, X. Lu and J. Fu, Industrial & Engineering Chemistry Research, 2019, 58, 15425-15431.
16. K. Chen, X. Zhang, T. Williams, L. Bourgeois and D. R. MacFarlane, Electrochimica Acta, 2017, 239, 84-89.
